# COVID-19 risk perception, preventive behaviors, vaccination acceptance and hesitancy, and psychosocial status in liver transplant recipients

**DOI:** 10.3389/fpubh.2026.1884917

**Published:** 2026-09-04

**Authors:** Sami Akbulut, Nazlican Bagci, Ayse Baran, Mustafa Arslan, Kemal Baris Sarici, Ertugrul Karabulut, Zeynep Burcin Yilmaz

**Affiliations:** 1 Department of Surgery and Liver Transplantation, Inonu University Faculty of Medicine, Malatya, Türkiye; 2 Department of Public Health, Inonu University Faculty of Medicine, Malatya, Türkiye; 3 Department of Biostatistics and Medical Informatics, Inonu University Faculty of Medicine, Malatya, Türkiye; 4 Department of Surgical Nursing, Inonu University Faculty of Nursing, Malatya, Türkiye; 5 Department of Nursing Service, Inonu University Faculty of Medicine, Malatya, Türkiye; 6 Department of Infectious Diseases and Clinical Microbiology, Inonu University Faculty of Medicine, Malatya, Türkiye

**Keywords:** COVID-19, liver transplantation, preventive behavior, psychological well-being, risk perception, vaccination

## Abstract

**Background:**

Liver transplant (LT) recipients may experience substantial clinical and psychosocial vulnerability during infectious disease outbreaks because of lifelong immunosuppression. This study evaluated COVID-19 vaccination uptake, vaccine acceptance and hesitancy, preventive behavior, risk perception, and psychosocial well-being among LT recipients.

**Methods:**

This cross-sectional analytical study included 311 adult LT recipients followed at a tertiary liver transplantation institute in Türkiye. Data were collected through structured face-to-face interviews using a standardized questionnaire and three validated instruments: the Short Depression–Happiness Scale (SDHS), COVID-19 Prevention Guidelines Compliance Scale (COVID-PGCS), and COVID-19 Risk Perception Scale (COVID-RPS). Exploratory group comparisons, correlation analyses, and multivariable linear regression models were performed.

**Results:**

COVID-19 vaccination uptake was 84.9%. The most frequently reported reasons for vaccination were recommendations from health professionals, perceived higher mortality among patients with chronic diseases, high vaccine effectiveness, and recommendations from health authorities. The leading reasons for vaccine hesitancy were insufficient evidence regarding vaccine effectiveness and safety, anti-vaccination messages on social media, and concerns about adverse effects. In multivariable analyses, higher SDHS scores were independently associated with middle income (*p* = 0.006), high income (*p* = 0.021), everolimus use (*p* = 0.041), and influence of scientific journals and official documents (*p* = 0.006), whereas COVID-19 infection after BNT162b2 vaccination was associated with lower SDHS scores (*p* = 0.004). The latter estimate should be interpreted cautiously because this subgroup included only seven recipients. No individual variable was independently associated with COVID-PGCS scores. In the full COVID-RPS model, middle income was independently associated with lower scores (*p* = 0.011), whereas motivation to protect parents and children was associated with higher scores (*p* = 0.040). COVID-19 vaccination status and the infection-timing categories were not statistically significant in the full model. Sensitivity analyses showed that vaccination status became significant when infection timing was excluded (*p* = 0.009), whereas infection before vaccination became significant when vaccination status was excluded (*p* = 0.006), indicating model-specification dependence.

**Conclusion:**

COVID-19 vaccination uptake was high among LT recipients. Psychosocial well-being and risk perception were associated with selected socioeconomic, clinical, and informational factors, whereas no individual variable examined was independently associated with preventive compliance. Vaccination status and infection timing should not be interpreted as robust, mutually independent determinants of COVID-19 risk perception. These findings support reliable scientific communication, psychosocial support, and sustained engagement with transplant recipients during future infectious disease outbreaks.

## 1 Introduction

COVID-19 was first identified in December 2019 in Wuhan, Hubei Province, China, and was later designated coronavirus disease 2019 by the World Health Organization (WHO) during the investigation of a cluster of pneumonia cases. The WHO officially declared COVID-19 a pandemic on March 11, 2020 (1, 2). COVID-19 is caused by severe acute respiratory syndrome coronavirus 2 (SARS-CoV-2), a single-stranded, positive-sense RNA virus with an enveloped structure characterized by crown-like spike glycoproteins (3, 4). As of January 1, 2026, more than 778 million confirmed COVID-19 cases and over 7.1 million deaths had been reported to the WHO worldwide. However, a recent cohort-based epidemiological modeling study estimated that the actual global COVID-19 mortality burden may have reached 11.3 million deaths (95% uncertainty interval, 7.8–17.4 million), substantially exceeding the officially reported mortality (5, 6). As of mid-2026, Türkiye had reported 17,004,726 confirmed COVID-19 cases and 101,419 deaths to the WHO, corresponding to a crude case-fatality ratio of 0.60% (7). These statistics highlight the global burden of the pandemic and regional variations in its impact (8). Beyond its epidemiological burden, COVID-19 disrupted social and economic systems and reshaped individual risk perception and health-related behaviors, particularly among clinically vulnerable populations (9, 10). Vaccination programs were central components of the public-health response to COVID-19, although their uptake and deployment were influenced by healthcare-system capacity, communication strategies, and access-related factors (11). Vaccination status may also be associated with risk perception and adherence to preventive behaviors (9).

Most SARS-CoV-2 infections are asymptomatic or mild to moderate; however, clinical management depends on disease severity and the individual risk of progression, with early antiviral treatment recommended for selected high-risk patients (2, 12, 13). However, the risk of morbidity and mortality increases in older adults and in individuals with preexisting conditions, including cardiovascular disease, diabetes, immunosuppression, chronic respiratory disease, and cancer (14–16). As immunocompromised individuals, liver transplant (LT) recipients are particularly vulnerable to infectious diseases because of their underlying liver disease history and post-transplant immunosuppressive therapy (17). In addition to increased clinical susceptibility, LT recipients may experience heightened psychological stress and behavioral adaptations during large-scale public health emergencies (18, 19).

Since the first successful LT performed by Starzl et al. in 1967, LT has become the gold standard treatment for numerous liver diseases, including acute and chronic liver conditions (20). Following LT surgery, LT recipients are prescribed various immunosuppressive drug regimens, which may differ across medical centers. These regimens include calcineurin inhibitors (tacrolimus, cyclosporine), antimetabolites (mycophenolate, azathioprine), corticosteroids, and mTOR inhibitors (everolimus, sirolimus) (21). Although these therapies are essential for preventing organ rejection, they also increase susceptibility to metabolic complications, malignancies, and opportunistic infections (21–23). These complications can negatively affect quality of life and emotional well-being, potentially limiting social interaction and shaping infection-related risk perception and preventive behavior among LT recipients.

Against the background of widespread concerns regarding severe COVID-19 outcomes among older and immunocompromised individuals, LT recipients faced considerable uncertainty about their clinical vulnerability during the pandemic. Survey studies involving LT and other solid organ transplant recipients documented frequent fear of infection, heightened perceived risk, greater risk-avoidant behavior, and stronger adherence to protective measures. Although LT recipients also reported greater perceived loneliness and social isolation, their overall psychological well-being was not consistently worse than that of nontransplant controls (19, 24).

Evidence regarding COVID-19-related mortality in LT recipients has similarly remained heterogeneous. Early systematic reviews reported substantial case-fatality rates among infected transplant recipients. Fraser et al. (25) reported a COVID-19-related mortality rate of approximately 19.3% among LT recipients, whereas Nacif et al. (26) reported an overall mortality rate of 25.6% among solid organ transplant recipients and a rate of 37.5% in the small LT subgroup. In contrast, subsequent comparative cohorts and systematic reviews suggested that LT status was not consistently associated with higher mortality than that observed in the general population (25, 27–29). Studies conducted at our center likewise reported lower COVID-19-related mortality than that observed in the general population in an overall LT cohort and low observed mortality among recipients who underwent LT for primary liver cancer, although the latter finding was derived from a specific and relatively small subgroup (30, 31). Collectively, these divergent findings suggest that COVID-19 outcomes among LT recipients may be shaped not only by LT status but also by age, comorbidity burden, vaccination status, immunosuppressive treatment, pandemic phase, and differences in case selection.

Despite the growing literature on clinical outcomes, considerably less is known about how LT recipients perceived COVID-19 risk and how these perceptions were associated with preventive behavior and psychosocial well-being. Available evidence suggests that infection-related concerns may encourage protective behavior, while lockdowns and social-distancing measures may simultaneously create difficulties in accessing routine transplant care and contribute to social disruption (19, 24, 32). These interconnected clinical, psychological, and behavioral dimensions underscore the need to evaluate risk perception, preventive compliance, and psychosocial status together in LT recipients.

COVID-19-related mortality is associated with a range of demographic and clinical factors, including male sex, advanced age, smoking, obesity, diabetes mellitus, renal disease, chronic liver disease, pulmonary disease, hypertension, cerebrovascular disease, and immune dysfunction (33–35). Given their lifelong exposure to immunosuppressive therapy and frequent comorbidity burden, LT recipients represent a clinically vulnerable population whose responses to infectious disease outbreaks warrant particular attention. However, clinical vulnerability alone does not fully explain how recipients perceive infection-related risks, adopt preventive measures, make vaccination decisions, or experience the psychological consequences of a pandemic. Evidence addressing these interconnected dimensions among LT recipients remains limited, particularly with regard to the factors associated with risk perception, preventive compliance, vaccination acceptance and hesitancy, and psychosocial status. Accordingly, this cross-sectional analytical study aimed to evaluate COVID-19 vaccination uptake, vaccine acceptance- and hesitancy-related factors, risk perception, adherence to preventive measures, and psychosocial status among LT recipients, as well as the factors associated with these outcomes.

## 2 Materials and methods

### 2.1 Study design, setting, and period

This cross-sectional analytical study evaluated COVID-19 risk perception, compliance with preventive measures, vaccination-related attitudes, and psychosocial status among LT recipients. Following ethical approval, data were collected between February and June 2023 through structured face-to-face interviews conducted during routine outpatient follow-up visits at the Inonu University Liver Transplant Institute, a tertiary referral center in Malatya, Türkiye. At our center, LT recipients are generally followed once a month during the first post-transplant year and every three months thereafter. The participants were attending these regularly scheduled post-transplant follow-up visits and were not specifically invited for COVID-19 surveillance. The study assessed their COVID-19-related perceptions and preventive behaviors, vaccination experiences, psychosocial status, and self-reported previous COVID-19 infection history; it did not involve prospective clinical monitoring for newly occurring COVID-19 infections.

### 2.2 Population and sample of the study

The source population comprised 1,550 adult LT recipients who underwent LT at our center between 2002 and 2021, were alive during the study period, and were native Turkish speakers or sufficiently proficient in Turkish. The required sample size was calculated using a 95% confidence level, a 5% margin of error, and a finite population size of 1,550, yielding a minimum required sample of 309 participants. During the data-collection period, adult LT recipients attending routine outpatient follow-up visits were consecutively screened for eligibility. Recipients who met the predefined inclusion criteria, had no exclusion criteria, and voluntarily agreed to participate were consecutively enrolled until the planned sample size was exceeded. Structured face-to-face interviews were conducted with 320 recipients by two members of the research team, MA and NB, using the standardized questionnaire. Nine participants were excluded because they did not complete all sections of the questionnaire, leaving 311 recipients in the final analytic cohort.

### 2.3 Inclusion and exclusion criteria

LT recipients aged 18 years or older who could communicate effectively in Turkish, understand the questionnaire items, and had been discharged from inpatient care following transplantation were included in the study. Clinically stable patients who regularly attended postoperative follow-up visits and voluntarily agreed to participate after being informed about the study objectives were enrolled. Conversely, patients who were not proficient in Turkish, had cognitive or communication limitations preventing accurate responses, or did not complete the questionnaire were excluded. Likewise, those with severe medical complications or those who were hospitalized during data collection were not included in the study.

### 2.4 Parameters and scales used in the study

#### 2.4.1 Demographic and clinical characteristics form

The questionnaire used in this study consisted of 16 items and three validated scales designed to comprehensively assess sociodemographic, psychological, and behavioral characteristics. Accordingly, data were collected and analyzed on participants’ age, sex, educational attainment, marital status, monthly household income, employment status, and the presence of chronic comorbidities. Clinical variables included underlying primary liver disease—namely hepatitis B virus (HBV), hepatitis C virus (HCV), hepatocellular carcinoma (HCC), cryptogenic cirrhosis, acute fulminant liver failure, autoimmune liver disease, Budd–Chiari syndrome, alcohol-related liver disease, primary sclerosing cholangitis (PSC), Wilson disease, primary biliary cholangitis (PBC), giant hemangioma, and metabolic dysfunction–associated steatohepatitis (MASH)—as well as type of liver transplantation (living-donor liver transplantation [LDLT] or deceased-donor liver transplantation [DDLT]). Additional variables included chronic comorbidities, namely diabetes mellitus (DM), hypertension (HT), cardiovascular disease (CVD), osteoporosis, chronic obstructive pulmonary disease (COPD), and chronic kidney disease (CKD). Furthermore, immunosuppressive medications used after liver transplantation—tacrolimus, everolimus, prednisolone, mycophenolate mofetil (MMF), and cyclosporine A (CsA)—were recorded. Vaccination-related data included COVID-19 vaccination status and COVID-19 vaccination regimen, categorized as CoronaVac only (Sinovac Life Sciences), BNT162b2 only (Pfizer–BioNTech), heterologous vaccination, or unvaccinated. Heterologous vaccination was defined as having received at least one dose of both CoronaVac and BNT162b2. Reasons for vaccination and factors contributing to vaccine hesitancy were also recorded.

#### 2.4.2 Short Depression-Happiness Scale (SDHS)

The SDHS was developed by Joseph et al. in 2004 (36) and adapted into Turkish by Sapmaz and Temizel in 2013 (37). It is a 4-point Likert-type (0 = never, 1 = rarely, 2 = sometimes, 3 = often) instrument comprising six items—three positive and three negative statements. Negative items are reverse-coded, and higher total scores indicate greater happiness, whereas lower scores denote increased depressive tendencies. The Turkish version demonstrated good internal consistency (Cronbach’s alpha = 0.80).

#### 2.4.3 COVID-19 Risk Perception Scale (COVID-RPS)

The COVID-RPS was developed by Plohl and Musil in 2021 (38) and validated in Turkish by Karatana et al. (39). It measures individuals’ perceived risk related to the COVID-19 pandemic. It contains six items on a 7-point Likert scale (1 = strongly disagree to 7 = strongly agree). The first four items are intended to assess contamination perception (perceived risk of becoming infected), whereas the final two items aim to evaluate fear perception related to COVID-19. Accordingly, in the present study, COVID-RPS results are reported separately as total score, contamination perception score, and fear perception score. Higher scores reflect greater perceived risk. The Turkish version exhibited acceptable reliability (Cronbach’s alpha = 0.79) (39).

#### 2.4.4 COVID-19 Prevention Guidelines Compliance Scale (COVID-PGCS)

The COVID-PGCS was also developed by Plohl and Musil in 2021 (38) and adapted into Turkish by Karatana et al. in 2021 (39). It evaluates adherence to preventive behaviors recommended by the WHO and the Centers for Disease Control and Prevention (CDC). This 4-point Likert-type scale includes 11 items (1 = not at all to 4 = to a great extent), with higher scores indicating better compliance with COVID-19 protection measures. The Turkish version showed strong internal consistency (Cronbach’s alpha = 0.94) (39).

### 2.5 Study protocol, ethical considerations, and funding

This study was conducted in accordance with the ethical principles of the 1964 Declaration of Helsinki and its subsequent amendments and complied with all applicable institutional and national regulations governing research involving human participants. Ethical approval was obtained from the Inonu University Scientific Research Ethics Committee for Health Sciences (Approval Date: 24 January 2023; Approval Number: 4443). No financial support was received for the design, conduct, participant recruitment, data collection, statistical analysis, interpretation of the findings, or preparation of the manuscript. In 2026, financial support was obtained solely to cover the article processing charge for this publication from the Inonu University Scientific Research Projects Coordination Unit (Project ID: TSA-2026-4593). The study was designed, conducted, analyzed, and reported in accordance with the STROBE (Strengthening the Reporting of Observational Studies in Epidemiology) guidelines to enhance transparency, reproducibility, and methodological rigor (40). All participants were informed about the study, and both verbal and written informed consent were obtained prior to participation.

### 2.6 Statistical analysis

All statistical analyses were performed using IBM SPSS Statistics for Windows, version 25.0 (IBM Corp., Armonk, NY, USA). The distribution of continuous variables was assessed using the Kolmogorov–Smirnov test. Continuous variables with an approximately normal distribution were presented as mean ± standard deviation (SD), together with 95% confidence intervals (CIs) for the mean, whereas categorical variables were summarized as frequencies and percentages. Comparisons between two independent groups were performed using Student’s t-test, and comparisons among three or more independent groups were conducted using one-way analysis of variance (ANOVA). The homogeneity of variance assumption was evaluated using Levene’s test. When the overall ANOVA result was statistically significant, Bonferroni-adjusted *post hoc* tests were used for pairwise comparisons. Comparisons between categorical variables were performed using the chi-square test. The internal consistency reliability of the study scales was evaluated using Cronbach’s alpha coefficient. The univariate comparisons were considered exploratory and hypothesis-generating, and no global multiplicity correction was applied across these analyses. For subgroup comparisons involving vaccine acceptance– and hesitancy-related items, inferential tests and 95% CIs were not calculated when either comparison group included fewer than 10 participants or when no participant represented the relevant category. These results were presented descriptively and indicated by an em dash (—) in the tables.

Multiple linear regression analyses were performed to identify factors independently associated with the SDHS total score, COVID-PGCS total score, and total COVID-RPS score. For each outcome, variables with a *p*-value < 0.10 in the univariate analyses and/or variables considered clinically relevant were entered simultaneously into the multivariable models using the enter method. Multicategory categorical predictors were represented by binary indicator (dummy) variables before inclusion in the regression models, with one category designated as the reference category. COVID-19 infection timing was coded as a mutually exclusive five-category variable applicable to the entire cohort, with “never diagnosed with COVID-19” as the reference category. Cases with missing data for any variable included in a given model were excluded using listwise deletion. All three final regression models included the complete analytic cohort of 311 participants.

Multicollinearity was assessed using tolerance values and variance inflation factors (VIFs), with VIF values < 5 considered acceptable. Model performance was evaluated using the coefficient of determination (R^2^), adjusted R^2^, and the standard error of the estimate, while the overall statistical significance of each model was assessed using the F-statistic. Unstandardized regression coefficients (B), standard errors (SEs), standardized regression coefficients (*β*), 95% CIs for B, and *p*-values were reported. To assess the potential overlap between COVID-19 vaccination status and infection timing in the total COVID-RPS model, two sensitivity analyses were performed: one excluding vaccination status and the other excluding the infection-timing categories. Additional sensitivity analyses were performed by entering sex as a forced covariate into each of the three multivariable models. Correlations between scale scores were assessed using Spearman’s rho because the scores were derived from Likert-type items and showed minor deviations from normality. All statistical tests were two-sided, and a p-value < 0.05 was considered statistically significant.

## 3 Results

### 3.1 General characteristics of the study population

Table 1 summarizes the sociodemographic and clinical characteristics of the 311 LT recipients. The age distribution showed that 43 patients (13.8%) were ≤35 years, 42 (13.5%) were aged 36–45 years, 69 (22.2%) were aged 46–55 years, 119 (38.3%) were aged 56–65 years, and 38 (12.2%) were ≥66 years. The majority of recipients were male (76.5%), while 23.5% of patients were female. Most participants were married (85.2%), and 46 (14.8%) were unmarried. Regarding educational attainment, 21 recipients (6.8%) had no formal education, 128 (41.2%) had completed primary education, 49 (15.8%) had completed lower secondary education, 76 (24.4%) had completed upper secondary education, and 37 (11.9%) had completed tertiary education. Monthly household income was reported as low income in 145 patients (46.6%), middle income in 147 (47.3%), and high income in 19 (6.1%). The majority of recipients were unemployed (*n* = 265, 85.2%), whereas 46 (14.8%) were employed.

**Table 1:** Sociodemographic and clinical characteristics of liver transplant recipients.

Variable	Category	n	%
Age group	≤35 years	43	13.8
	36–45 years	42	13.5
	46–55 years	69	22.2
	56–65 years	119	38.3
	≥66 years	38	12.2
Sex	Male	238	76.5
	Female	73	23.5
Educational attainment	No formal	21	6.8
	Primary	128	41.2
	Lower secondary	49	15.8
	Upper secondary	76	24.4
	Tertiary	37	11.9
Marital Status	Married	265	85.2
	Unmarried	46	14.8
Monthly household income	Low income	145	46.6
	Middle income	147	47.3
	High income	19	6.1
Employment status	Employed	46	14.8
	Unemployed	265	85.2
Etiology of liver disease	HBV	143	46.0
	Cryptogenic cirrhosis	49	15.8
	HBV + HCC	40	12.9
	HCC with other etiologies	18	5.8
	Acute (fulminant) liver failure	14	4.5
	HCV	9	2.9
	Autoimmune	7	2.3
	Budd–Chiari syndrome	7	2.3
	Alcohol-related liver disease	7	2.3
	PSC	6	1.9
	Wilson	4	1.3
	PBC	3	1.0
	Giant Hemangioma	2	0.6
	MASH	2	0.6
Type of LT	DDLT	28	9.0
	LDLT	283	91.0
Presence of chronic comorbidity	Present	109	35.0
	Absent	202	65.0
Specific comorbidities	DM	71	22.8
	HT	40	12.9
	CVD	15	4.8
	Osteoporosis	8	2.6
	COPD	3	1.0
	CKD	4	1.3
	Other conditions	16	5.1
Post-transplant immunosuppressive therapy	Tacrolimus (Received)	302	97.1
	Everolimus (Received)	127	40.8
	Prednisolone (Received)	74	23.8
	MMF (Received)	72	23.2
	CsA (Received)	1	0.3

CVD, cardiovascular disease; CKD, chronic kidney disease; COPD, chronic obstructive pulmonary disease; CsA, cyclosporine A; DDLT, deceased donor liver transplantation; DM, diabetes mellitus; HBV, hepatitis B virus; HCC, hepatocellular carcinoma; HCV, hepatitis C virus; HT, hypertension; LDLT, living donor liver transplantation; LT, liver transplantation; MASH, metabolic dysfunction–associated steatohepatitis; MMF, mycophenolate mofetil; PBC, primary biliary cholangitis; PSC, primary sclerosing cholangitis.

The most common etiology of primary liver disease was HBV–related liver disease (46.0%), followed by cryptogenic cirrhosis (15.8%) and HBV–associated HCC (12.9%). LDLT was performed in 283 recipients (91.0%), while 28 patients (9.0%) underwent DDLT. Chronic comorbidities were present in 109 recipients (35.0%) and absent in 202 (65.0%). DM (22.8%) and HT (12.9%) were the most frequently reported comorbid conditions. Post-transplant immunosuppressive therapy included tacrolimus in 302 recipients (97.1%), everolimus in 127 (40.8%), prednisolone in 74 (23.8%), MMF in 72 (23.2%), and CsA in 1 patient (0.3%).

### 3.2 COVID-19 vaccination status and SARS-CoV-2 exposure

Table 2 summarizes the COVID-19 vaccination coverage and SARS-CoV-2 exposure status of LT recipients. A total of 264 recipients (84.9%) received at least one COVID-19 vaccine dose, whereas 47 (15.1%) were unvaccinated. Among vaccinated recipients (*n* = 264), 102 (38.6%) received CoronaVac only, 87 (33.0%) received BNT162b2 only, and 75 (28.4%) received heterologous vaccination. A total of 104 recipients (33.4%) had previously been diagnosed with COVID-19, while 207 (66.6%) had never been diagnosed with COVID-19. Among those who had COVID-19 (*n* = 104), 43 patients (41.3%) were infected before vaccination, 30 (28.8%) after CoronaVac vaccination, 7 (6.7%) after BNT162b2 vaccination, and 24 (23.1%) were unvaccinated individuals with prior COVID-19 infection.

**Table 2:** COVID-19 history, vaccination status, and vaccine acceptance and hesitancy among liver transplant recipients.

COVID-19 vaccination status, history, and related factors	*n*	%
COVID-19 vaccination status		
Vaccinated	264	84.9
Unvaccinated	47	15.1
COVID-19 vaccination regimen (% calculated among vaccinated patients only)		
CoronaVac only	102	38.6
BNT162b2 only	87	33.0
Heterologous vaccination	75	28.4
Unvaccinated	47	—
Have you ever been diagnosed with COVID-19?		
Yes, I have	104	33.4
No, I have not	207	66.6
COVID-19 infection relative to vaccination (% diagnosed patients only)		
Before vaccination	43	41.3
After CoronaVac vaccination	30	28.8
Unvaccinated individuals with prior COVID-19 infection	24	23.1
After BNT162b2 vaccination	7	6.7
Never diagnosed with COVID-19	207	—
Factors influencing COVID-19 vaccine acceptance *		
Recommendations from health professionals	225	72.3
Higher COVID-19 mortality in patients with chronic diseases	192	61.7
High vaccine effectiveness	171	55.0
Recommendations from the Ministry of Health and health authorities	168	54.0
Protecting my parents and children	64	20.6
Preventing disease transmission and protecting others	53	17.0
Recommendations from vaccinated family members or friends	30	9.6
Recommendations from individuals previously infected with COVID-19	16	5.1
Influence of scientific journals and official documents	13	4.2
Requirement for access to schools or workplaces	11	3.5
Access to commercial centers and public transportation	8	2.6
Trust in vaccine-producing countries	7	2.3
No specific reason	2	0.6
Factors contributing to COVID-19 vaccine hesitancy *		
Insufficient evidence on vaccine effectiveness and safety	77	24.8
Anti-vaccination messages on social media	68	21.9
Concerns about vaccine side effects reported in studies	50	16.1
Concerns about the profit motives of vaccine manufacturers	15	4.8
Belief that vaccine components may cause autism or other disorders	4	1.3
Belief that vaccines may cause infertility	4	1.3
Belief that children’s immune systems are immature and vaccines are harmful	2	0.6
Belief that natural infection provides stronger immunity than vaccination	1	0.3
Complementary and alternative medicine is effective with fewer side effects	0	0.0

Percentages for vaccination regimens were calculated among vaccinated recipients, and percentages for infection timing were calculated among recipients previously diagnosed with COVID-19. Unvaccinated and never-diagnosed categories are shown for completeness and were not included in these percentage calculations. *More than one option is marked.

The most frequently reported factors influencing COVID-19 vaccine acceptance were recommendations from health professionals (*n* = 225, 72.3%), higher COVID-19 mortality in patients with chronic diseases (*n* = 192, 61.7%), high vaccine effectiveness (*n* = 171, 55.0%), and recommendations from the Ministry of Health and health authorities (*n* = 168, 54.0%). Other reported factors influencing COVID-19 vaccine acceptance included protecting my parents and children (*n* = 64, 20.6%), preventing disease transmission and protecting others (*n* = 53, 17.0%), recommendations from vaccinated family members or friends (*n* = 30, 9.6%), recommendations from individuals previously infected with COVID-19 (*n* = 16, 5.1%), influence of scientific journals and official documents (*n* = 13, 4.2%), requirement for access to schools or workplaces (*n* = 11, 3.5%), access to commercial centers and public transportation (*n* = 8, 2.6%), and trust in vaccine-producing countries (*n* = 7, 2.3%).

Factors contributing to COVID-19 vaccine hesitancy included insufficient evidence on vaccine effectiveness and safety (*n* = 77, 24.8%), anti-vaccination messages on social media (*n* = 68, 21.9%), concerns about vaccine side effects reported in studies (*n* = 50, 16.1%), concerns about the profit motives of vaccine manufacturers (*n* = 15, 4.8%), belief that vaccine components may cause autism or other disorders (*n* = 4, 1.3%), belief that vaccines may cause infertility (*n* = 4, 1.3%), belief that natural infection provides stronger immunity than vaccination (*n* = 1, 0.3%), and belief that children’s immune systems are immature and vaccines are harmful (*n* = 2, 0.6%). No participants reported complementary and alternative medicine as a contributing factor (0%).

### 3.3 Psychological well-being assessed by SDHS Scale

Table 3 presents SDHS total scores across demographic, clinical, immunosuppressive, vaccination-related, and COVID-19–related variables. The mean SDHS score of the study population was 13.8 ± 4.3 (95% CI, 13.3–14.2). SDHS scores did not differ significantly across the remaining demographic, clinical, immunosuppressive, vaccination-related, and COVID-19–related variables.

**Table 3:** Distribution of SDHS scores according to sociodemographic and clinical characteristics in liver transplant recipients.

Variable	Category	Mean	SD	95% CI	*p*
Age group	≤35 years	12.7	5.0	11.1–14.2	0.471
	36–45 years	13.8	3.8	12.6–15.0	
	46–55 years	14.1	4.4	13.0–15.1	
	56–65 years	14.0	4.0	13.2–14.7	
	≥66 years	13.7	4.5	12.2–15.1	
Sex	Male	13.9	4.2	13.4–14.5	0.180
	Female	13.2	4.5	12.1–14.2	
Educational attainment	No formal	14.2	3.7	12.5–15.9	0.567
	Primary	13.7	4.2	13.0–14.5	
	Lower secondary	13.6	4.1	12.4–14.7	
	Upper secondary	13.3	4.8	12.3–14.4	
	Tertiary	14.7	3.9	13.4–16.0	
Marital Status	Married	13.9	4.2	13.4–14.4	0.270
	Unmarried	13.1	4.9	11.7–14.6	
Monthly household income	Low income	12.9	4.8	12.1–13.7	**0.001**
	Middle income	14.4	3.8	13.7–15.0	
	High income	15.8	2.1	14.8–16.8	
Employment status	Employed	14.4	3.5	13.3–15.4	0.308
	Unemployed	13.7	4.4	13.1–14.2	
Presence of chronic comorbidity	Present	13.9	3.7	13.2–14.6	0.576
	Absent	13.7	4.6	13.0–14.3	
HT	Present	14.3	3.1	13.3–15.3	0.366
	Absent	13.7	4.4	13.1–14.2	
DM	Present	13.8	3.8	12.9–14.7	0.861
	Absent	13.7	4.4	13.2–14.3	
CVD	Present	13.3	3.2	11.5–15.0	0.653
	Absent	13.8	4.3	13.3–14.3	
Everolimus	Received	14.4	3.9	13.7–15.0	**0.037**
	Not received	13.3	4.5	12.7–14.0	
MMF	Received	13.9	4.4	12.9–14.9	0.711
	Not received	13.7	4.3	13.2–14.3	
Prednisolone	Received	13.9	4.0	13.0–14.9	0.702
	Not received	13.7	4.4	13.1–14.3	
COVID-19 vaccination status	Vaccinated	13.8	4.3	13.3–14.3	0.571
	Unvaccinated	13.4	4.4	12.1–14.7	
COVID-19 vaccination regimen	CoronaVac only	13.7	4.4	12.8–14.5	0.568
	BNT162b2 only	13.6	4.2	12.7–14.5	
	Heterologous vaccination	14.3	4.3	13.3–15.2	
Have you ever been diagnosed with COVID-19?	Yes, I have	13.8	4.1	13.0–14.6	0.939
	No, I have not	13.7	4.4	13.1–14.3	
COVID-19 infection relative to vaccination	Before vaccination	14.6	3.6	13.5–15.7	**0.009**
	After CoronaVac vaccination	13.8	4.4	12.1–15.4	
	After BNT162b2 vaccination	9.0	3.7	5.6–12.4	
	Unvaccinated individuals with prior COVID-19 infection	13.7	3.9	12.1–15.4	

BNT162b2, Pfizer–BioNTech COVID-19 vaccine; CVD, cardiovascular disease; CI, confidence interval; COVID-19, Coronavirus disease 2019; CoronaVac, inactivated SARS-CoV-2 vaccine (Sinovac); DM, diabetes mellitus; HT, hypertension; LT, liver transplantation; MMF, mycophenolate mofetil; SD, standard deviation; SDHS, Short Depression–Happiness Scale.Bold values indicate statistical significance (*p* < 0.05).

In exploratory univariate analyses, significant differences in SDHS scores were observed according to monthly household income (*p* = 0.001), everolimus use (*p* = 0.037), and COVID-19 infection relative to vaccination (*p* = 0.009). *Post hoc* pairwise comparisons with Bonferroni correction demonstrated that SDHS scores were significantly higher in the high-income group compared with the low-income group (mean difference = 2.9, *p* = 0.015) and significantly higher in the middle-income group compared with the low-income group (mean difference = 1.5, *p* = 0.010). According to COVID-19 infection relative to vaccination, Bonferroni-adjusted pairwise comparisons indicated that SDHS scores were significantly lower in LT recipients infected after BNT162b2 vaccination compared with those infected before vaccination (mean difference = 5.6, *p* = 0.004) and those infected after CoronaVac vaccination (mean difference = 4.8, *p* = 0.028).

Tables 4, 5 show the associations of vaccine acceptance– and hesitancy-related factors with SDHS scores. In these exploratory comparisons, higher SDHS scores were observed among recipients who reported influence of scientific journals and official documents (*p* < 0.001). Comparisons involving trust in vaccine-producing countries and the belief that children’s immune systems are immature and vaccines are harmful were presented descriptively only because the affirmative-response groups contained fewer than 10 participants.

**Table 4:** Relationship between vaccine acceptance–related factors and SDHS scores.

Variable	Category	Mean	SD	95% CI	*p*
Recommendations from health professionals	Yes	13.8	4.2	13.2–14.3	0.889
	No	13.7	4.5	12.7–14.7	
Higher COVID-19 mortality in patients with chronic diseases	Yes	13.9	4.3	13.3–14.5	0.558
	No	13.6	4.3	12.8–14.4	
High vaccine effectiveness	Yes	14.0	4.4	13.3–14.7	0.239
	No	13.4	4.1	12.8–14.1	
Recommendations from the Ministry of Health and health authorities	Yes	13.7	4.3	13.0–14.3	0.703
	No	13.9	4.3	13.1–14.6	
Protecting my parents and children	Yes	14.1	4.1	13.1–15.1	0.475
	No	13.7	4.4	13.1–14.2	
Preventing disease transmission and protecting others	Yes	14.4	3.6	13.4–15.4	0.259
	No	13.6	4.4	13.1–14.2	
Recommendations from vaccinated family members or friends	Yes	13.8	4.3	12.2–15.4	0.949
	No	13.8	4.3	13.2–14.3	
Recommendations from individuals previously infected with COVID-19	Yes	13.1	3.8	11.0–15.1	0.509
	No	13.8	4.3	13.3–14.3	
Influence of scientific journals and official documents	Yes	16.9	1.8	15.8–18.0	**<0.001**
	No	13.6	4.3	13.1–14.1	
Requirement for access to schools or workplaces	Yes	14.0	3.3	11.8–16.2	0.846
	No	13.7	4.3	13.3–14.2	
Access to commercial centers and public transportation	Yes	12.8	5.0	—	—
	No	13.8	4.3	—	
Trust in vaccine-producing countries	Yes	16.6	1.8	—	—
	No	13.7	4.3	—	
No specific reason	Yes	11.0	9.9	—	—
	No	13.8	4.3	—	

Bold values indicate statistical significance (*p* < 0.05).

**Table 5:** Relationship between vaccine hesitancy–related beliefs and SDHS scores.

Variable	Category	Mean	SD	95% CI	*p*
Insufficient evidence on vaccine effectiveness and safety	Yes	14.0	3.8	13.2–14.9	0.539
	No	13.7	4.4	13.1–14.2	
Anti-vaccination messages on social media	Yes	13.2	4.3	12.1–14.2	0.210
	No	13.9	4.3	13.4–14.5	
Concerns about vaccine side effects reported in studies	Yes	14.1	4.0	12.9–15.2	0.580
	No	13.7	4.4	13.2–14.2	
Concerns about the profit motives of vaccine manufacturers	Yes	11.6	5.6	8.5–14.7	0.147
	No	13.9	4.2	13.4–14.3	
Belief that vaccine components may cause autism or other disorders	Yes	15.8	2.9	—	—
	No	13.7	4.3	—	
Belief that vaccines may cause infertility	Yes	12.8	3.9	—	—
	No	13.8	4.3	—	
Belief that children’s immune systems are immature and vaccines are harmful	Yes	4.0	0.0	—	—
	No	13.8	4.2	—	
Belief that natural infection provides stronger immunity than vaccination	Yes	15.0	—	—	—
	No	13.8	4.3	—	
Complementary and alternative medicine is effective with fewer side effects	Yes	—	—	—	—
	No	13.8	4.3	—	

— indicates fewer than 10 participants in the subgroup or no participants in the corresponding category; inferential statistics and 95% confidence intervals were therefore not reported.

**Figure 1:**
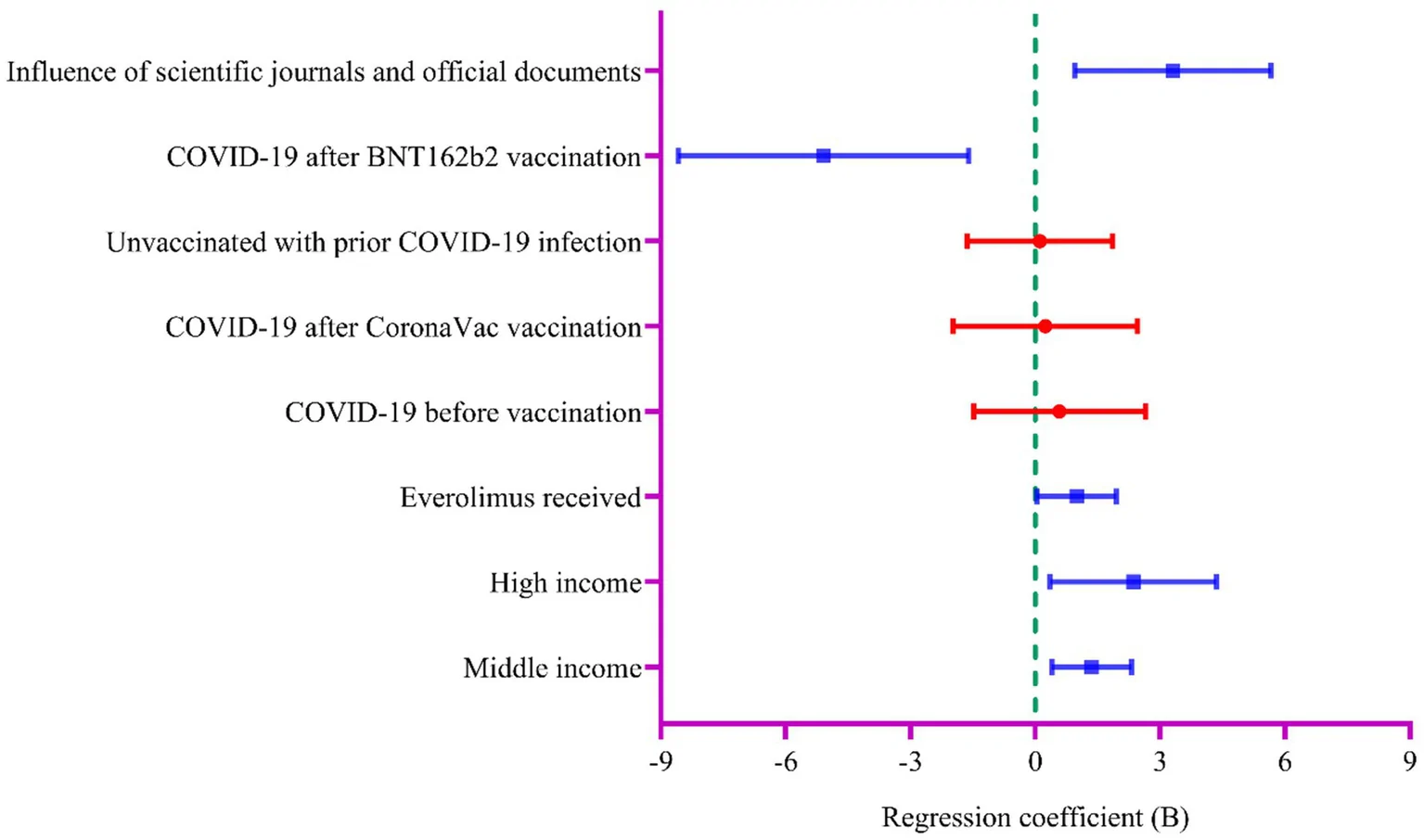
Forest plot of multivariable-adjusted unstandardized regression coefficients (*B*) and 95% confidence intervals for SDHS scores. The vertical dashed line indicates the null value (*B* = 0). Reference categories were low income, no everolimus use, never diagnosed with COVID-19, and no reported influence of scientific journals or official documents. The estimate for COVID-19 infection after BNT162b2 vaccination should be interpreted cautiously because this category included only seven participants.

Table 6 and Figure 1 present the multivariable linear regression analysis of factors associated with SDHS scores in the complete analytic cohort (*n* = 311). Compared with the low-income group, middle income (*p* = 0.006, VIF = 1.097) and high income (*p* = 0.021, VIF = 1.093) were significantly associated with higher SDHS scores. Everolimus use was also associated with higher SDHS scores compared with no everolimus use (*p* = 0.041, VIF = 1.047). Reporting influence from scientific journals and official documents was associated with higher SDHS scores compared with reporting no such influence (*p* = 0.006, VIF = 1.063). In contrast, COVID-19 infection after BNT162b2 vaccination was associated with lower SDHS scores compared with never having been diagnosed with COVID-19 (*p* = 0.004, VIF = 1.278). COVID-19 infection among unvaccinated individuals (*p* = 0.905, VIF = 3.250), infection before vaccination (*p* = 0.580, VIF = 2.424), and infection after CoronaVac vaccination (*p* = 0.833, VIF = 2.046) were not significantly associated with SDHS scores. Collinearity diagnostics indicated no problematic multicollinearity, with tolerance values ranging from 0.308 to 0.955 and VIF values ranging from 1.047 to 3.250. The overall model was statistically significant, *F*(8, 302) = 4.515, *p* < 0.001, and explained 10.7% of the variance in SDHS scores (R^2^ = 0.107; adjusted R^2^ = 0.083). The standard error of the estimate was 4.10. Because only seven participants were included in the COVID-19 infection after BNT162b2 vaccination category, this estimate should be interpreted cautiously.

**Table 6:** Multivariable linear regression analysis of factors associated with SDHS scores.

Variable	Category	*B*	SE	*β*	*p*	95% CI for *B*
Monthly household income (Reference: low income)	Middle income	1.36	0.49	0.16	0.006	0.39 to 2.32
	High income	2.35	1.02	0.13	0.021	0.36 to 4.35
Everolimus (Reference: not received)	Received	0.99	0.48	0.11	0.041	0.04 to 1.95
COVID-19 infection relative to vaccination (Reference: never diagnosed with COVID-19)	Before vaccination	0.58	1.05	0.05	0.580	−1.48 to 2.65
	After CoronaVac vaccination	0.24	1.13	0.02	0.833	−1.98 to 2.46
	Unvaccinated individuals with prior COVID-19 infection	0.11	0.89	0.01	0.905	−1.64 to 1.86
	After BNT162b2 vaccination	−5.09	1.77	−0.18	0.004	−8.58 to −1.60
Influence of scientific journals and official documents (Reference: no)	Yes	3.31	1.20	0.16	0.006	0.95 to 5.66
Constant	—	12.37	0.91	—	<0.001	10.58 to 14.15

B, unstandardized regression coefficient; SE, standard error; *β*, standardized regression coefficient; CI, confidence interval; Analytic *n* = 311; overall model: F(8,302) = 4.515, *p* < 0.001.

### 3.4 Preventive behavior and guideline compliance assessed by COVID-PGCS Scale

Table 7 presents COVID-PGCS scores across study variables. The mean COVID-PGCS score was 37.8 ± 5.4 (95% CI, 37.2–38.4). COVID-PGCS scores did not differ significantly across the remaining demographic, clinical, vaccination-related, or COVID-19–related variables. In exploratory univariate analyses, significant differences in COVID-PGCS scores were observed according to age group (*p* = 0.048), hypertension status (*p* = 0.029), and COVID-19 vaccination regimen (*p* = 0.038). Although the overall comparison across age groups reached statistical significance (*p* = 0.048), *post hoc* pairwise comparisons with Bonferroni correction did not identify statistically significant differences between individual age categories, suggesting a modest overall effect rather than distinct group-level contrasts. LT recipients with hypertension had higher COVID-PGCS scores than those without hypertension (mean, 39.2 vs. 37.6). According to the COVID-19 vaccination regimen, Bonferroni-adjusted pairwise comparisons demonstrated that recipients with heterologous vaccination had significantly higher COVID-PGCS scores than those who received BNT162b2 only (*p* = 0.033).

**Table 7:** COVID-PGCS scores across sociodemographic and clinical subgroups of liver transplant recipients.

Variable	Category	Mean	SD	95% CI	p
Age group	≤35 years	36.6	6.6	34.6–38.7	**0.048**
	36–45 years	38.0	4.4	36.6–39.4	
	46–55 years	36.9	6.0	35.5–38.4	
	56–65 years	37.9	5.0	37.0–38.8	
	≥66 years	39.8	3.8	38.6–41.1	
Sex	Male	37.6	5.5	36.9–38.3	0.405
	Female	38.2	4.7	37.1–39.3	
Educational attainment	No formal	36.9	3.7	35.2–38.6	0.527
	Primary	37.7	5.3	36.7–38.6	
	Lower secondary	37.1	5.2	35.7–38.6	
	Upper secondary	38.0	6.1	36.6–39.4	
	Tertiary	39.0	4.9	37.3–40.6	
Marital Status	Married	37.9	5.2	37.3–38.6	0.179
	Unmarried	36.8	6.3	34.9–38.6	
Monthly household income	Low income	37.9	4.8	37.1–38.6	0.273
	Middle income	37.5	5.9	36.5–38.4	
	High income	39.5	4.1	37.5–41.5	
Employment status	Employed	37.2	5.6	35.5–38.8	0.403
	Unemployed	37.9	5.3	37.2–38.5	
Presence of chronic comorbidity	Present	38.4	4.6	37.5–39.2	0.150
	Absent	37.4	5.7	36.6–38.2	
HT	Present	39.2	4.0	37.9–40.4	**0.029**
	Absent	37.6	5.5	36.9–38.2	
DM	Present	38.2	4.6	37.1–39.3	0.436
	Absent	37.6	5.6	36.9–38.3	
CVD	Present	38.5	5.4	35.5–41.5	0.602
	Absent	37.7	5.4	37.1–38.3	
Everolimus	Received	37.9	5.5	37.0–38.9	0.679
	Not received	37.7	5.3	36.9–38.4	
MMF	Received	37.6	6.0	36.2–39.0	0.805
	Not received	37.8	5.1	37.2–38.5	
Prednisolone	Received	37.3	6.0	35.9–38.7	0.393
	Not received	37.9	5.1	37.3–38.6	
COVID-19 vaccination status	Vaccinated	37.8	5.4	37.2–38.5	0.727
	Unvaccinated	37.5	5.2	36.0–39.0	
COVID-19 vaccination regimen	CoronaVac only	37.9	5.2	36.9–38.9	**0.038**
	BNT162b2 only	36.7	6.0	35.5–38.0	
	Heterologous vaccination	38.9	4.7	37.8–40.0	
Have you ever been diagnosed with COVID-19?	Yes, I have	37.2	5.7	36.0–38.3	0.156
	No, I have not	38.1	5.2	37.4–38.8	
COVID-19 infection relative to vaccination	Before vaccination	36.8	6.2	34.9–38.7	0.491
	After CoronaVac vaccination	38.4	5.2	36.5–40.4	
	After BNT162b2 vaccination	35.3	4.1	31.5–39.1	
	Unvaccinated individuals with prior COVID-19 infection	36.7	5.9	34.2–39.2	

BNT162b2: Pfizer–BioNTech COVID-19 vaccine; CVD: Cardiovascular disease; CI: Confidence interval; COVID-19: Coronavirus disease 2019; CoronaVac: Inactivated SARS-CoV-2 vaccine (Sinovac); DM, diabetes mellitus; HT, hypertension; LT, liver transplantation; MMF: Mycophenolate mofetil; SD: Standard deviation; COVID-19 PGCS: COVID-19 Prevention Guidelines Compliance Scale. Bold values indicate statistical significance (*p* < 0.05).

Tables 8, 9 show the associations of vaccine acceptance– and hesitancy-related factors with COVID-PGCS scores. In these exploratory comparisons, higher COVID-PGCS scores were observed among recipients who reported being influenced by recommendations from vaccinated family members or friends (*p* = 0.033) and those who indicated preventing disease transmission and protecting others as vaccine acceptance–related factors (*p* = 0.041). In contrast, vaccine hesitancy–related beliefs did not show consistent associations with COVID-PGCS scores.

**Table 8:** Relationship between vaccine acceptance–related factors and COVID-PGCS scores.

Variable	Category	Mean	SD	95% CI	*p*
Recommendations from health professionals	Yes	37.9	5.5	37.2–38.6	0.401
	No	37.4	5.0	36.3–38.4	
Higher COVID-19 mortality in patients with chronic diseases	Yes	38.2	5.2	37.4–38.9	0.108
	No	37.1	5.5	36.1–38.1	
High vaccine effectiveness	Yes	37.9	5.5	37.1–38.7	0.630
	No	37.6	5.2	36.7–38.5	
Recommendations from the Ministry of Health and health authorities	Yes	38.0	5.3	37.2–38.8	0.458
	No	37.5	5.5	36.6–38.4	
Protecting my parents and children	Yes	38.0	4.6	36.8–39.1	0.730
	No	37.7	5.5	37.0–38.4	
Preventing disease transmission and protecting others	Yes	39.1	4.3	38.0–40.3	**0.041**
	No	37.5	5.5	36.8–38.2	
Recommendations from vaccinated family members or friends	Yes	39.3	3.7	37.9–40.7	**0.033**
	No	37.6	5.5	37.0–38.2	
Recommendations from individuals previously infected with COVID-19	Yes	39.1	5.1	36.3–41.8	0.315
	No	37.7	5.4	37.1–38.3	
Influence of scientific journals and official documents	Yes	39.9	4.2	37.4–42.5	0.137
	No	37.7	5.4	37.1–38.3	
Requirement for access to schools or workplaces	Yes	37.1	4.7	33.9–40.3	0.673
	No	37.8	5.4	37.2–38.4	
Access to commercial centers and public transportation	Yes	40.1	3.0	—	—
	No	37.7	5.4	—	
Trust in vaccine-producing countries	Yes	36.7	3.7	—	—
	No	37.8	5.4	—	
No specific reason	Yes	41.5	3.5	—	—
	No	37.7	5.4	—	

— indicates fewer than 10 participants in the subgroup or no participants in the corresponding category; inferential statistics and 95% confidence intervals were therefore not reported. Bold values indicate statistical significance (*p* < 0.05).

**Table 9:** Relationship between vaccine hesitancy–related beliefs and COVID-PGCS scores.

Variable	Category	Mean	SD	95% CI	p
Insufficient evidence on vaccine effectiveness and safety	Yes	38.3	5.2	37.1–39.5	0.311
	No	37.6	5.4	36.9–38.3	
Anti-vaccination messages on social media	Yes	37.2	5.4	35.8–38.5	0.285
	No	37.9	5.3	37.3–38.6	
Concerns about vaccine side effects reported in studies	Yes	38.4	5.6	36.8–40.0	0.389
	No	37.7	5.3	37.0–38.3	
Concerns about the profit motives of vaccine manufacturers	Yes	36.5	5.7	33.4–39.7	0.363
	No	37.8	5.3	37.2–38.4	
Belief that vaccine components may cause autism or other disorders	Yes	33.3	5.0	—	—
	No	37.8	5.3	—	
Belief that vaccines may cause infertility	Yes	34.0	7.0	—	—
	No	37.8	5.3	—	
Belief that children’s immune systems are immature and vaccines are harmful	Yes	44.0	0.0	—	—
	No	37.7	5.4	—	
Belief that natural infection provides stronger immunity than vaccination	Yes	29.0	—	—	—
	No	37.8	5.3	—	
Complementary and alternative medicine is effective with fewer side effects	Yes	—	—	—	—
	No	37.8	5.4	—	

— indicates fewer than 10 participants in the subgroup or no participants in the corresponding category; inferential statistics and 95% confidence intervals were therefore not reported.

**Figure 2:**
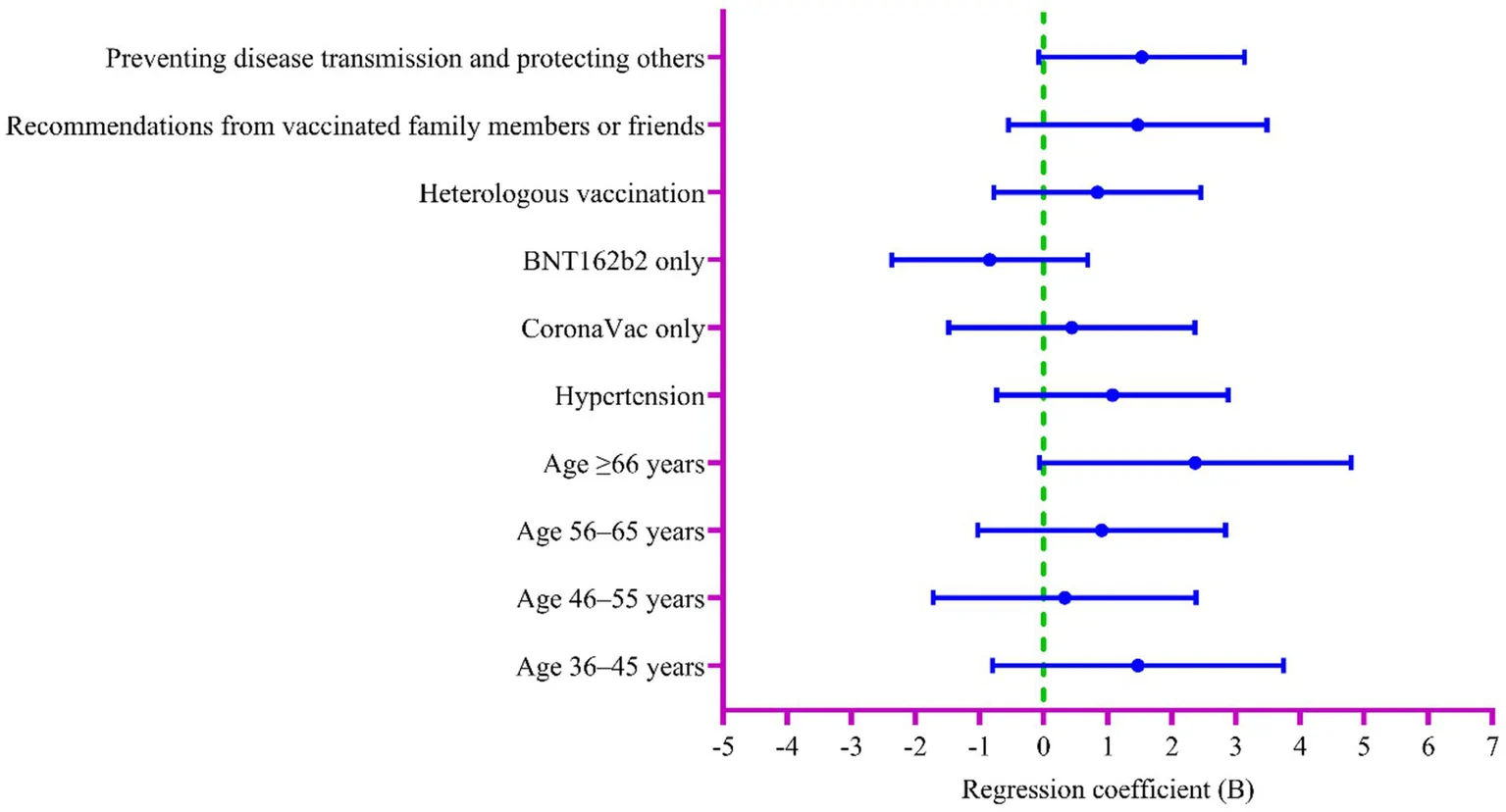
Forest plot of multivariable-adjusted unstandardized regression coefficients (*B*) and 95% confidence intervals for COVID-PGCS scores. The vertical dashed line indicates the null value (*B* = 0). Reference categories were age ≤35 years, no hypertension, unvaccinated status, no recommendation from vaccinated family members or friends, and no endorsement of preventing disease transmission and protecting others. All individual 95% confidence intervals crossed zero, indicating that no individual predictor reached statistical significance.

Table 10 and Figure 2 present the multivariable linear regression analysis of factors associated with COVID-PGCS scores in the complete analytic cohort (*n* = 311). None of the individual predictors included in the model was statistically significantly associated with COVID-PGCS scores at the conventional significance level. Compared with recipients aged ≤35 years, recipients aged 36–45 years (*p* = 0.204, VIF = 1.749), 46–55 years (*p* = 0.753, VIF = 2.102), 56–65 years (*p* = 0.359, VIF = 2.559), and ≥66 years (*p* = 0.057, VIF = 1.839) had higher estimated COVID-PGCS scores, although none of these differences reached statistical significance. Participants who endorsed preventing disease transmission and protecting others also had higher estimated COVID-PGCS scores, but this association did not reach statistical significance (*p* = 0.062, VIF = 1.055). Hypertension (*p* = 0.244, VIF = 1.061), CoronaVac-only vaccination (*p* = 0.655, VIF = 1.373), BNT162b2-only vaccination (*p* = 0.278, VIF = 1.365), heterologous vaccination (*p* = 0.308, VIF = 1.381), and recommendations from vaccinated family members or friends (*p* = 0.154, VIF = 1.030) were also not statistically significantly associated with COVID-PGCS scores. Collinearity diagnostics indicated no problematic multicollinearity, with tolerance values ranging from 0.391 to 0.971 and VIF values ranging from 1.030 to 2.559. The overall model was statistically significant, *F*(10, 300) = 2.079, *p* = 0.026, and explained 6.5% of the variance in COVID-PGCS scores (R^2^ = 0.065; adjusted R^2^ = 0.034). The standard error of the estimate was 5.26.

**Table 10:** Multivariable linear regression analysis of factors associated with COVID-PGCS scores.

Variable	Category	*B*	SE	*β*	*p*	95% CI for B
Age group (Reference: ≤35 years)	36–45 years	1.47	1.16	0.09	0.204	−0.80 to 3.74
	46–55 years	0.33	1.04	0.03	0.753	−1.72 to 2.38
	56–65 years	0.90	0.98	0.08	0.359	−1.03 to 2.84
	≥66 years	2.37	1.24	0.15	0.057	−0.07 to 4.80
Hypertension (Reference: absent)	Present	1.07	0.92	0.07	0.244	−0.74 to 2.88
COVID-19 vaccination regimen (Reference: Unvaccinated)	CoronaVac only	0.44	0.98	0.03	0.655	−1.48 to 2.36
	BNT162b2 only	−0.84	0.78	−0.07	0.278	−2.37 to 0.69
	Heterologous vaccination	0.84	0.82	0.07	0.308	−0.78 to 2.45
Recommendations from vaccinated family members or friends (Reference: No)	Yes	1.47	1.03	0.08	0.154	−0.55 to 3.48
Preventing disease transmission and protecting others (Reference: No)	Yes	1.53	0.82	0.11	0.062	−0.07 to 3.13
Constant	—	36.28	0.97	—	<0.001	34.37 to 38.20

*B*, unstandardized regression coefficient; SE, standard error; *β*, standardized regression coefficient; CI, confidence interval; analytic *n* = 311; overall model: F(10,300) = 2.079, *p* = 0.026.

### 3.5 Risk perception related to COVID-19 assessed by COVID-RPS Scale

Table 11 presents total COVID-RPS scores across study variables. The mean total COVID-RPS score was 28.9 ± 5.0, the mean COVID-RPS contamination perception score was 18.1 ± 2.9 (95% CI, 17.8–18.4), and the mean COVID-RPS fear perception score was 10.8 ± 2.9 (95% CI, 10.4–11.1). Total COVID-RPS scores did not differ significantly across the remaining demographic, clinical, vaccination-related, or COVID-19–related variables.

**Table 11:** Total COVID-RPS scores across sociodemographic, clinical, immunosuppressive, vaccination-related, and COVID-19–related variables.

Variable	Category	Mean	SD	95% CI	p
Age group	≤35 years	28.8	5.9	27.0–30.6	0.283
	36–45 years	29.8	4.2	28.5–31.1	
	46–55 years	27.8	4.9	26.6–29.0	
	56–65 years	29.2	5.2	28.2–30.1	
	≥66 years	29.0	4.5	27.5–30.4	
Sex	Male	28.6	5.1	28.0–29.3	0.115
	Female	29.7	4.8	28.6–30.8	
Educational attainment	No formal	27.8	4.5	25.7–29.8	0.089
	Primary	28.7	4.9	27.8–29.5	
	Lower secondary	29.9	4.6	28.6–31.3	
	Upper secondary	28.1	5.6	26.8–29.4	
	Tertiary	30.3	4.8	28.7–31.9	
Marital Status	Married	29.0	4.8	28.4–29.6	0.182
	Unmarried	28.0	6.0	26.2–29.7	
Monthly household income	Low income	29.6	4.6	28.9–30.4	**0.001**
	Middle income	27.8	5.2	27.0–28.7	
	High income	31.2	5.2	28.7–33.7	
Employment Status	Employed	27.6	4.3	26.3–28.9	0.061
	Unemployed	29.1	5.1	28.5–29.7	
Presence of chronic comorbidity	Present	29.4	4.6	28.5–30.3	0.171
	Absent	28.6	5.2	27.9–29.3	
HT	Present	30.1	4.2	28.8–31.4	0.098
	Absent	28.7	5.1	28.1–29.3	
DM	Present	29.2	4.8	28.0–30.3	0.590
	Absent	28.8	5.1	28.1–29.4	
CVD	Present	30.1	5.6	27.0–33.1	0.347
	Absent	28.8	5.0	28.2–29.4	
Everolimus	Received	28.2	4.5	27.4–29.0	**0.035**
	Not received	29.4	5.3	28.6–30.1	
MMF	Received	28.1	6.0	26.7–29.5	0.117
	Not received	29.1	4.7	28.5–29.7	
Prednisolone	Received	27.7	5.2	26.5–28.9	**0.025**
	Not received	29.2	5.0	28.6–29.9	
COVID-19 vaccination status	Vaccinated	28.8	5.1	28.2–29.4	0.469
	Unvaccinated	29.4	5.0	27.9–30.8	
COVID-19 vaccination regimen	CoronaVac only	28.4	5.2	27.3–29.4	0.327
	BNT162b2 only	28.7	5.0	27.6–29.7	
	Heterologous vaccination	29.5	4.9	28.4–30.6	
Have you ever been diagnosed with COVID-19?	Yes, I have	27.8	4.7	26.9–28.7	**0.009**
	No, I have not	29.4	5.1	28.7–30.1	
COVID-19 infection relative to vaccination	Before vaccination	26.1	4.7	24.6–27.5	**0.017**
	After CoronaVac vaccination	29.1	4.6	27.4–30.8	
	After BNT162b2 vaccination	29.1	2.0	27.3–31.0	
	Unvaccinated individuals with prior COVID-19 infection	28.9	4.8	26.8–30.9	

BNT162b2: Pfizer–BioNTech COVID-19 vaccine; CVD, cardiovascular disease; CI, confidence interval; COVID-19, Coronavirus disease 2019; CoronaVac: Inactivated SARS-CoV-2 vaccine (Sinovac); DM, diabetes mellitus; HT, hypertension; LT: Liver transplantation; MMF, mycophenolate mofetil; SD, standard deviation; COVID-19 RPS: COVID-19 Risk Perception Scale. Bold values indicate statistical significance (*p* < 0.05).

In exploratory univariate analyses, significant differences in total COVID-RPS scores were observed according to monthly household income (*p* = 0.001), everolimus use (*p* = 0.035), prednisolone use (*p* = 0.025), previous COVID-19 diagnosis (*p* = 0.009), and COVID-19 infection relative to vaccination (*p* = 0.017). *Post hoc* pairwise comparisons with Bonferroni correction demonstrated that total COVID-RPS scores were significantly higher in the low-income group compared with the middle-income group (*p* = 0.005) and significantly higher in the high-income group compared with the middle-income group (*p* = 0.017). In addition, total COVID-RPS scores were significantly lower in recipients who received everolimus compared with those who did not receive it (*p* = 0.035) and in those who received prednisolone compared with those who did not receive it (*p* = 0.025). Recipients previously diagnosed with COVID-19 also had lower total COVID-RPS scores than those who had never been diagnosed with COVID-19 (*p* = 0.009). Bonferroni-adjusted pairwise comparison further demonstrated that recipients infected before vaccination had significantly lower total COVID-RPS scores than those infected after CoronaVac vaccination (*p* = 0.034).

Supplementary Table S1 shows that, according to the contamination perception subscale, no significant differences were observed with respect to age group, sex, marital status, employment status, chronic comorbidity, DM, HT, CVD, COVID-19 vaccination status, COVID-19 vaccination regimen, or COVID-19 infection relative to vaccination. However, contamination perception scores differed significantly according to educational attainment (*p* = 0.019), monthly household income (*p* < 0.001), everolimus use (*p* = 0.037), prednisolone use (*p* = 0.021), and previous COVID-19 diagnosis (*p* = 0.008), with lower scores observed in recipients previously diagnosed with COVID-19. Bonferroni-adjusted pairwise comparisons indicated that contamination perception scores were significantly lower in the middle-income group than in the low-income group (mean difference = 1.0, *p* = 0.007) and significantly higher in the high-income group than in the middle-income group (mean difference = 2.3, *p* = 0.003).

Supplementary Table S2 shows that, regarding the fear perception subscale, significant differences were observed according to sex (*p* = 0.030), monthly household income (*p* = 0.035), employment status (*p* = 0.019), and hypertension status (*p* = 0.014). In addition, fear perception scores differed significantly according to COVID-19 infection relative to vaccination (*p* = 0.039). However, Bonferroni-adjusted *post hoc* comparisons did not identify any statistically significant pairwise differences between the categories of COVID-19 infection relative to vaccination. Bonferroni-adjusted pairwise comparisons indicated that fear perception scores were significantly lower in the middle-income group than in the low-income group (mean difference = 0.8, *p* = 0.048). No significant differences were detected for age group, educational attainment, marital status, chronic comorbidity, DM, CVD, immunosuppressive medication use, COVID-19 vaccination status, or COVID-19 vaccination regimen.

Tables 12, 13 show the associations between vaccine acceptance–related factors, vaccine hesitancy–related beliefs, and COVID-19 risk perception. According to Table 12, total COVID-RPS scores were significantly higher among recipients who reported recommendations from individuals previously infected with COVID-19 (*p* = 0.021), influence of scientific journals and official documents (*p* = 0.007), preventing disease transmission and protecting others (*p* = 0.004), and protecting my parents and children (*p* < 0.001). The comparison involving access to commercial centers or public transportation was presented descriptively only because the affirmative-response group contained fewer than 10 participants. According to Table 13, anti-vaccination messages on social media were nominally associated with lower COVID-19 risk perception (*p* = 0.049).

**Table 12:** Relationship between vaccine acceptance–related factors and total COVID-RPS scores.

Variable	Category	Mean	SD	95% CI	*p*
Recommendations from health professionals	Yes	28.9	4.9	28.2–29.5	0.939
	No	28.9	5.3	27.8–30.1	
Higher COVID-19 mortality in patients with chronic diseases	Yes	29.1	5.0	28.4–29.8	0.290
	No	28.5	5.1	27.6–29.4	
High vaccine effectiveness	Yes	28.6	5.2	27.8–29.4	0.231
	No	29.3	4.8	28.5–30.1	
Recommendations from the Ministry of Health and health authorities	Yes	28.9	4.9	28.1–29.6	0.989
	No	28.9	5.2	28.0–29.7	
Protecting my parents and children	Yes	30.9	4.6	29.8–32.1	**<0.001**
	No	28.3	5.0	27.7–29.0	
Preventing disease transmission and protecting others	Yes	30.7	5.1	29.3–32.1	**0.004**
	No	28.5	5.0	27.9–29.1	
Recommendations from vaccinated family members or friends	Yes	29.7	4.6	28.0–31.5	0.325
	No	28.8	5.1	28.2–29.4	
Recommendations from individuals previously infected with COVID-19	Yes	31.7	3.6	29.8–33.6	**0.021**
	No	28.7	5.1	28.1–29.3	
Influence of scientific journals and official documents	Yes	32.5	3.8	30.2–34.9	**0.007**
	No	28.7	5.0	28.1–29.3	
Requirement for access to schools or workplaces	Yes	30.6	4.1	27.8–33.3	0.262
	No	28.8	5.1	28.2–29.4	
Access to commercial centers and public transportation	Yes	32.8	3.4	—	—
	No	28.8	5.0	—	
Trust in vaccine-producing countries	Yes	31.9	3.8	—	—
	No	28.8	5.0	—	
No specific reason	Yes	33.5	0.7	—	—
	No	28.8	5.0	—	

— indicates fewer than 10 participants in the subgroup or no participants in the corresponding category; inferential statistics and 95% confidence intervals were therefore not reported. Bold values indicate statistical significance (*p* < 0.05).

**Table 13:** Relationship between vaccine hesitancy–related beliefs and total COVID-RPS scores.

Variable	Category	Mean	SD	95% CI	*p*
Insufficient evidence on vaccine effectiveness and safety	Yes	29.3	5.0	28.1–30.4	0.421
	No	28.7	5.0	28.1–29.4	
Anti-vaccination messages on social media	Yes	27.8	4.6	26.7–28.9	**0.049**
	No	29.2	5.1	28.5–29.8	
Concerns about vaccine side effects reported in studies	Yes	29.2	5.2	27.7–30.7	0.615
	No	28.8	5.0	28.2–29.4	
Concerns about the profit motives of vaccine manufacturers	Yes	27.4	4.6	24.8–30.0	0.246
	No	29.0	5.1	28.4–29.5	
Belief that vaccine components may cause autism or other disorders	Yes	24.5	2.7	—	—
	No	28.9	5.0	—	
Belief that vaccines may cause infertility	Yes	25.8	1.7	—	—
	No	28.9	5.1	—	
Belief that children’s immune systems are immature and vaccines are harmful	Yes	34.5	0.7	—	—
	No	28.8	5.0	—	
Belief that natural infection provides stronger immunity than vaccination	Yes	27.0	—	—	—
	No	28.9	5.0	—	
Complementary and alternative medicine is effective with fewer side effects	Yes	—	—	—	—
	No	28.9	5.0	—	

— indicates fewer than 10 participants in the subgroup or no participants in the corresponding category; inferential statistics and 95% confidence intervals were therefore not reported. Bold values indicate statistical significance (*p* < 0.05).

**Figure 3:**
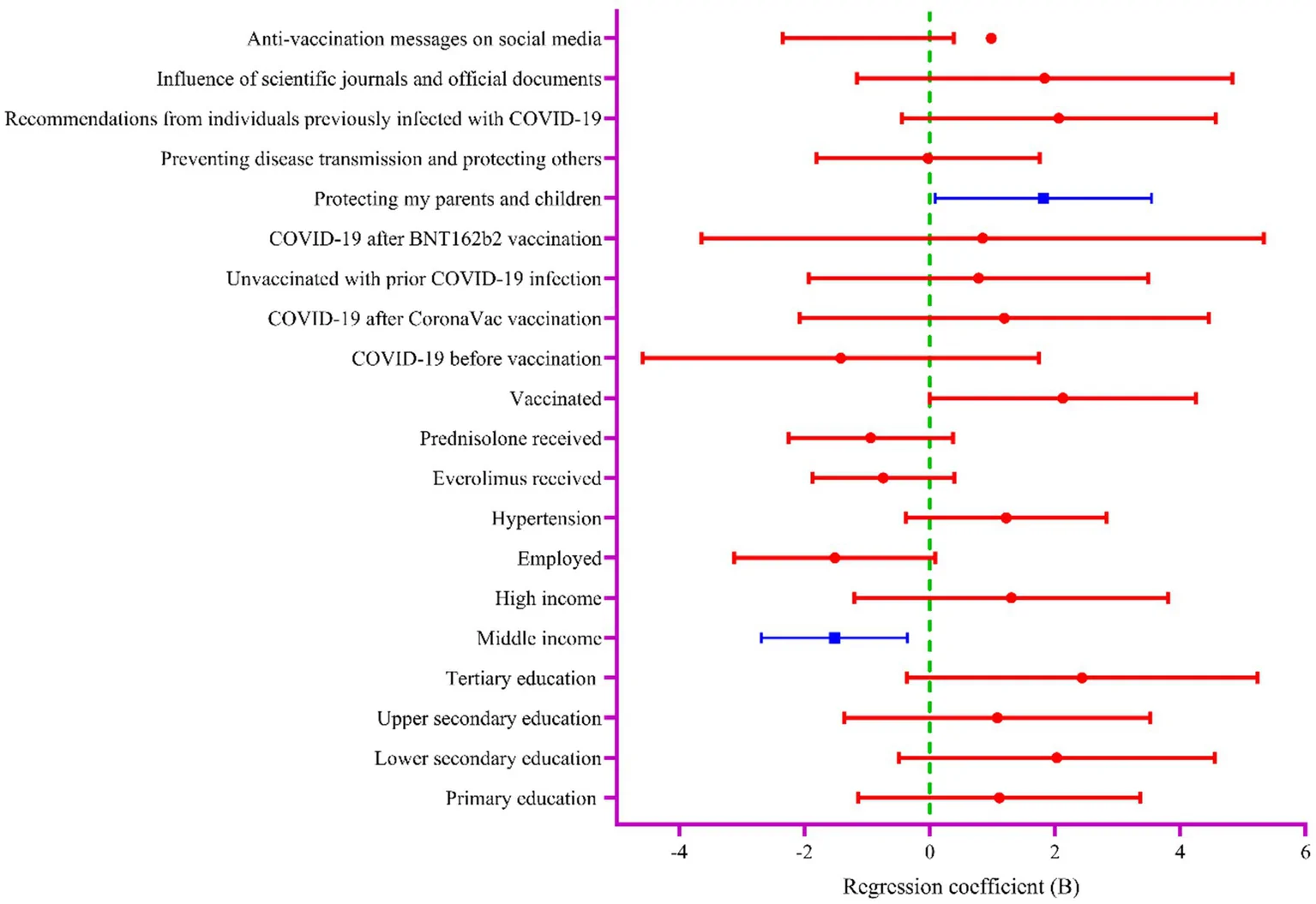
Forest plot of multivariable-adjusted unstandardized regression coefficients (*B*) and 95% confidence intervals from the full model for total COVID-RPS scores. The vertical dashed line indicates the null value (*B* = 0). Reference categories were no formal education, low income, unemployed status, no hypertension, no use of everolimus or prednisolone, unvaccinated status, never diagnosed with COVID-19, and a “No” response for each vaccine acceptance– and hesitancy-related item. Confidence intervals that do not cross zero indicate statistically significant associations.

Table 14 and Figure 3 present the multivariable linear regression analysis of factors associated with total COVID-RPS scores in the complete analytic cohort (*n* = 311). Compared with recipients with no formal education, primary education (*p* = 0.332, VIF = 4.518), lower secondary education (*p* = 0.115, VIF = 3.112), upper secondary education (*p* = 0.385, VIF = 4.057), and tertiary education (*p* = 0.088, VIF = 3.028) were not significantly associated with total COVID-RPS scores. Compared with the low-income group, middle income was significantly associated with lower total COVID-RPS scores (*p* = 0.011, VIF = 1.249), whereas high income was not significantly associated with the outcome (*p* = 0.306, VIF = 1.326). Employment status (*p* = 0.064, VIF = 1.194), hypertension (*p* = 0.135, VIF = 1.060), everolimus use (*p* = 0.198, VIF = 1.143), and prednisolone use (*p* = 0.159, VIF = 1.156) were not significantly associated with total COVID-RPS scores. COVID-19 vaccination status showed a borderline association but was not statistically significant according to the predefined *p* < 0.05 threshold (*p* = 0.050, VIF = 2.138). Compared with recipients who had never been diagnosed with COVID-19, infection before vaccination (*p* = 0.377, VIF = 4.392), infection after CoronaVac vaccination (*p* = 0.473, VIF = 3.429), prior infection among unvaccinated recipients (*p* = 0.572, VIF = 6.026), and infection after BNT162b2 vaccination (*p* = 0.711, VIF = 1.634) were not significantly associated with total COVID-RPS scores. Endorsement of protecting parents and children was significantly associated with higher total COVID-RPS scores (*p* = 0.040, VIF = 1.798). Preventing disease transmission and protecting others (*p* = 0.976, VIF = 1.657), recommendations from individuals previously infected with COVID-19 (*p* = 0.107, VIF = 1.131), influence of scientific journals and official documents (*p* = 0.230, VIF = 1.328), and anti-vaccination messages on social media (*p* = 0.159, VIF = 1.178) were not significantly associated with total COVID-RPS scores. Tolerance values ranged from 0.166 to 0.944. VIF values were below 5 for all predictors except previous COVID-19 infection among unvaccinated recipients (VIF = 6.026), indicating localized overlap in this category rather than generalized multicollinearity. The overall model was statistically significant, *F*(20, 290) = 3.539, *p* < 0.001, and explained 19.6% of the variance in total COVID-RPS scores (R^2^ = 0.196; adjusted R^2^ = 0.141). The standard error of the estimate was 4.67.

**Table 14:** Multivariable linear regression analysis of factors associated with total COVID-RPS scores.

Variable	Category	*B*	SE	*β*	*p*	95% CI for B
Educational attainment (Reference: no formal)	Primary	1.11	1.14	0.11	0.332	−1.14 to 3.36
	Lower secondary	2.03	1.28	0.15	0.115	−0.49 to 4.55
	Upper secondary	1.08	1.24	0.09	0.385	−1.36 to 3.52
	Tertiary	2.43	1.42	0.16	0.088	−0.37 to 5.23
Monthly household income (Reference: low income)	Middle income	−1.52	0.59	−0.15	0.011	−2.69 to −0.36
	High income	1.30	1.27	0.06	0.306	−1.20 to 3.81
Employment status (Reference: unemployed)	Employed	−1.52	0.81	−0.11	0.064	−3.12 to 0.09
Hypertension (Reference: absent)	Present	1.22	0.81	0.08	0.135	−0.38 to 2.82
Everolimus (Reference: not received)	Received	−0.74	0.58	−0.07	0.198	−1.88 to 0.39
Prednisolone (Reference: not received)	Received	−0.94	0.67	−0.08	0.159	−2.26 to 0.37
COVID-19 vaccination status (Reference: unvaccinated)	Vaccinated	2.12	1.08	0.15	0.050	−0.002 to 4.25
COVID-19 infection relative to vaccination (Reference: never diagnosed with COVID-19)	Before vaccination	−1.42	1.61	−0.10	0.377	−4.58 to 1.74
	After CoronaVac vaccination	1.19	1.66	0.07	0.473	−2.07 to 4.46
	Unvaccinated individuals with prior COVID-19 infection	0.78	1.38	0.07	0.572	−1.93 to 3.49
	After BNT162b2 vaccination	0.85	2.28	0.03	0.711	−3.64 to 5.33
Protecting my parents and children (Reference: no)	Yes	1.81	0.88	0.15	0.040	0.09 to 3.54
Preventing disease transmission and protecting others (Reference: no)	Yes	−0.03	0.91	0.00	0.976	−1.81 to 1.76
Recommendations from individuals previously infected with COVID-19 (Reference: no)	Yes	2.06	1.27	0.09	0.107	−0.45 to 4.57
Influence of scientific journals and official documents (Reference: no)	Yes	1.83	1.52	0.07	0.230	−1.17 to 4.83
Anti-vaccination messages on social media (Reference: no)	Yes	−0.98	0.70	−0.08	0.159	−2.35 to 0.39
Constant	—	27.66	1.75	—	<0.001	24.21 to 31.11

B, unstandardized regression coefficient; SE, standard error; *β*, standardized regression coefficient; CI, confidence interval; Analytic *n* = 311; overall model: F(20,290) = 3.539, *p* < 0.001. The binary variable indicating previous COVID-19 diagnosis was not entered separately because previous infection status was already represented by the categories of COVID-19 infection relative to vaccination. Previous COVID-19 diagnosis was not entered simultaneously with the infection-timing indicators because it was exactly determined by these indicators, resulting in perfect multicollinearity (tolerance = 0; VIF not estimable).

Two *post hoc* sensitivity analyses, both including all 311 participants, were performed to examine the overlap between COVID-19 vaccination status and infection timing. When vaccination status was excluded, the model remained statistically significant, *F*(19,291) = 3.487, *p* < 0.001, with R^2^ = 0.185 and adjusted R^2^ = 0.132. In this model, infection before vaccination was associated with lower total COVID-RPS scores (*B* = −3.43, SE = 1.25, *β* = −0.24, 95% CI: −5.88 to −0.98, *p* = 0.006). All VIF values were below 5, ranging from 1.059 to 4.501. When the infection-timing categories were excluded, the model was also statistically significant, *F*(16,294) = 3.875, *p* < 0.001, with R^2^ = 0.174 and adjusted R^2^ = 0.129. In this model, COVID-19 vaccination status was associated with higher total COVID-RPS scores (*B* = 2.13, SE = 0.81, *β* = 0.15, 95% CI: 0.54 to 3.71, *p* = 0.009). All VIF values were below 5, ranging from 1.047 to 4.489. The change in statistical significance across the alternative models indicated that the estimates for vaccination status and infection timing were sensitive to model specification and should not be interpreted as robust, mutually independent associations.

### 3.6 Scale Reliability and Inter-Scale correlations

Internal consistency varied across the study scales. The SDHS showed excellent internal consistency, with a Cronbach’s alpha coefficient of 0.936 and corrected item–total correlations ranging from 0.70 to 0.89. The COVID-PGCS also exhibited good reliability (Cronbach’s alpha = 0.899), with corrected item–total correlations between 0.40 and 0.84. In contrast, the COVID-RPS demonstrated moderate internal consistency, with a Cronbach’s alpha of 0.652 and corrected item–total correlations ranging from −0.07 to 0.62.

In exploratory Spearman correlation analyses, as expected, the total COVID-RPS score was strongly correlated with its contamination perception subscale (rho = 0.858, *p* < 0.001) and fear perception subscale (rho = 0.890, *p* < 0.001). A moderate positive correlation was observed between COVID-RPS contamination perception and fear perception (rho = 0.558, *p* < 0.001). Total COVID-RPS was positively correlated with COVID-PGCS at a weak-to-moderate level (rho = 0.333, *p* < 0.001). COVID-PGCS was more strongly correlated with fear perception (rho = 0.407, *p* < 0.001) than with contamination perception (rho = 0.203, *p* < 0.001). SDHS showed a weak positive correlation with COVID-PGCS (rho = 0.132, *p* = 0.020). No significant correlations were observed between SDHS and total COVID-RPS or COVID-RPS fear perception, whereas a weak negative correlation was observed between SDHS and COVID-RPS contamination perception (rho = −0.125, *p* = 0.028).

## 4 Discussion

This study examined COVID-19 vaccination uptake, vaccine acceptance and hesitancy, preventive behavior, risk perception, and psychosocial well-being among LT recipients. The findings showed a high vaccination uptake of 84.9%, with recommendations from health professionals, perceived higher mortality among patients with chronic diseases, high vaccine effectiveness, and recommendations from health authorities emerging as the main factors influencing vaccine acceptance. In the multivariable analyses, selected socioeconomic, clinical, infection-related, and informational factors were independently associated with SDHS and total COVID-RPS scores, whereas none of the individual variables included in the COVID-PGCS model reached statistical significance. Overall, these findings indicate that vaccination behavior, psychosocial well-being, preventive compliance, and risk perception in LT recipients are related but distinct domains shaped by different sets of factors.

Vaccination behavior, encompassing both vaccine uptake and hesitancy, is context dependent and shaped by interacting individual, social, informational, and structural determinants, including perceived vaccine benefits and risks, social norms and recommendations, access to reliable information, and institutional influences (41, 42). Vaccination is strongly recommended for LT recipients receiving long-term immunosuppressive therapy because of their increased risk of COVID-19–related morbidity and mortality (43–47). Regarding vaccine acceptance, Pan et al. (48) reported in a cross-sectional study of LT recipients in China that the leading reasons for willingness to receive a COVID-19 vaccine were confidence in vaccination as an effective preventive measure and concern about contracting COVID-19. Similarly, Chen et al. (49), in an internet-based survey of Chinese SOT recipients, found that willingness to receive a COVID-19 vaccine was independently associated with liver transplantation, obtaining vaccine information from physicians, scientists, or scientific journals, having at least a college-level education, intending to receive influenza vaccination during the current season, and perceiving vaccination as important for SOT recipients. In contrast, having received influenza vaccination during the previous season was independently associated with lower odds of willingness to receive a COVID-19 vaccine. Jackson et al. (50) reported high overall confidence in COVID-19 vaccine safety and efficacy among solid-organ transplant recipients, despite relatively low concern about contracting or transmitting COVID-19. Trust in physicians’ opinions and confidence in vaccine safety and protection against severe disease were among the most strongly endorsed attitudes. These findings suggest that confidence in medical guidance may have been particularly salient in this cohort, whereas fear-based concerns related to infection and transmission were less prominent. Consistent with this pattern, the most frequently reported factors influencing vaccine acceptance in the present study were recommendations from health professionals, perceived higher COVID-19 mortality among patients with chronic diseases, perceived high vaccine effectiveness, and recommendations from the Ministry of Health and other health authorities.

Vaccine hesitancy is a major global public health concern and has represented an important barrier to COVID-19 vaccination efforts. Studies conducted during the pandemic have suggested that COVID-19 vaccine hesitancy may have increased over time, although its prevalence varies across populations and settings (51, 52). In 2019, the WHO identified vaccine hesitancy as one of the ten major threats to global health (51, 53). Vaccine hesitancy has been defined as a delay in acceptance or refusal of vaccination despite the availability of vaccination services. Its determinants are context-specific and may include perceived disease risk, uncertainty regarding vaccine effectiveness and safety, concerns about adverse effects, and exposure to misinformation (52). Chen et al. (49) reported that the most frequently cited reasons for COVID-19 vaccine hesitancy among Chinese solid-organ transplant recipients were concerns related to pre-existing comorbidities, fear of vaccine adverse effects, and negative advice from physicians. Pan et al. (48) reported that the most frequently cited reasons for COVID-19 vaccine hesitancy among LT recipients were concerns about vaccine safety and adverse effects, potential interactions with current medications, possible effects on existing chronic diseases, and adverse effects on liver function. In multivariable analysis, vaccine hesitancy was associated with female sex, having a relative or friend with a medical background, not having received influenza vaccination during the previous season, having no intention to receive influenza vaccination during the current season, obtaining vaccine information primarily from healthcare workers, perceiving COVID-19 vaccination as inconvenient or unsafe after LT, not perceiving vaccination as important for LT recipients, lack of a recommendation from the transplant surgeon, and negative attitudes among family members or friends. Dey et al. (54), in a systematic review and meta-analysis of studies conducted in Indian populations, reported that the most frequently identified barriers to COVID-19 vaccine acceptance were fear of adverse effects, reported in 37% of the included studies, and concerns regarding vaccine efficacy and safety, each reported in 30%. Jackson et al. (50) reported that, among the nine respondents who were unwilling to receive a COVID-19 vaccine, hesitancy was attributed to perceived lack of efficacy, insufficient testing or evidence, and concerns about vaccine safety. Nevertheless, overall confidence in vaccine safety and efficacy was high in the full cohort, and 93.6% of respondents had already been vaccinated, indicating that vaccine hesitancy was uncommon among the survey respondents. Similar concerns were observed in the present study, in which insufficient evidence regarding vaccine effectiveness and safety, anti-vaccination messages on social media, and concerns about reported adverse effects were the most frequently cited contributors to vaccine hesitancy. Despite these concerns, 84.9% of LT recipients had received at least one COVID-19 vaccine dose, demonstrating high vaccine uptake within the study cohort. These findings highlight the relevance of trusted clinical guidance and reliable information sources in vaccine-related decision-making among LT recipients.

Beyond vaccination-related attitudes, risk perception is an important component of psychological and behavioral responses to infectious disease outbreaks. Previous studies have shown that risk perception, knowledge, and preventive behaviors are closely related to public responses and transmission dynamics during the COVID-19 pandemic. Higher perceived risk has generally been associated with greater adoption of preventive behaviors and compliance with public health recommendations, although it may also be associated with fear, anxiety, and psychological distress (55, 56). Clavel et al. (55), in a systematic scoping review, found that COVID-19 risk perception, knowledge, and preventive behaviors were generally moderate to high across general and high-risk adult populations. Several sociodemographic characteristics, including female sex, older age, higher education, and urban residence, were associated with greater knowledge and more consistent preventive behaviors. Cipolletta et al. (56), in a systematic review and qualitative synthesis, reported that higher COVID-19 risk perception was generally associated with greater adoption of preventive behaviors and adherence to social-distancing measures. The review identified associations between risk perception and compliance with public health recommendations, demographic and individual characteristics, geographical factors, and the timing of data collection during the pandemic. Moazzam et al. (57), in a cross-sectional study of solid-organ transplant recipients, reported high adherence to COVID-19 infection-control measures. Higher levels of COVID-19–related fear were associated with selected precautionary behaviors, including mask use, avoidance of in-person clinic visits, and leaving shoes outside the home, although the reported effect sizes were small. These findings suggest that greater perceived threat may be associated with increased engagement in some preventive behaviors among transplant recipients.

Drawing on the Health Belief Model and collective resilience theory, Herbas-Torrico and Frank (58) reported that COVID-19 preventive behaviors were associated with individual vulnerability, attitudes toward disease prevention, and social orientation. Chronic health problems showed a U-shaped association with preventive behavior, while older age and female sex were independently associated with greater adherence. Attitudes toward social distancing, lockdown measures, and their enforcement were also positively associated with preventive behaviors and showed some of the strongest associations in the model. Similarly, high levels of adherence to preventive measures have been reported in other high-risk clinical populations. In a cross-sectional study of adults with hypertension, 97.7% of participants reported regular face mask use, while 89.2% reported maintaining distance from people outside their household as a protective measure against COVID-19 (59). Beyond behavioral compliance, substantial psychological distress was reported during the COVID-19 outbreak. In a meta-analysis of 12 community-based studies, Bueno-Notivol et al. (60) found that the prevalence of depressive symptoms ranged from 7.45 to 48.30%, with a pooled prevalence of 25% (95% CI, 18–33%). The marked between-study heterogeneity was partly related to differences in the assessment instruments used. Previous reviews have identified female sex and age-related vulnerability among the demographic factors potentially associated with poorer mental health during pandemics, although COVID-19–specific evidence for older adults remains limited (61).

In a case–control study, Gastelum-Strozzi et al. (62) found that patients with rheumatic diseases reported greater perceived COVID-19 severity and likelihood of contagion, as well as more pronounced economic impact and several negative emotional responses, than age- and sex-matched controls. Furthermore, Herbas-Torrico and Frank (58) found that greater depressive emotional burden was independently associated with lower engagement in COVID-19 disease-prevention behaviors. The authors interpreted depression as a potential psychological barrier to preventive behavior, possibly through reduced self-efficacy and perceived ability to cope with the threat.

Within this broader context, COVID-PGCS scores were generally high among LT recipients in our cohort. In bivariate analyses, COVID-PGCS scores differed significantly according to age group, hypertension status, COVID-19 vaccination regimen, and the endorsement of preventing disease transmission and protecting others or recommendations from vaccinated family members or friends as vaccination-related influences. In the multivariable linear regression analysis, however, no single predictor was independently associated with COVID-PGCS scores at the predefined *p* < 0.05 threshold. Although the overall model was statistically significant (*p* = 0.026), its explanatory power was limited (R^2^ = 0.065; adjusted R^2^ = 0.034). Correlation analyses further showed that preventive compliance was associated with COVID-19 risk perception, particularly its fear dimension, and was weakly but significantly correlated with SDHS scores. Overall, preventive compliance showed significant bivariate relationships with perceived risk and psychosocial well-being, but no individual covariate retained a statistically significant independent association in the adjusted model.

In the present study, total COVID-RPS was positively correlated with COVID-PGCS at a weak-to-moderate level (rho = 0.333, *p* < 0.001). This relationship was stronger for the fear-perception dimension (rho = 0.407, *p* < 0.001) than for contamination perception (rho = 0.203, *p* < 0.001), suggesting that the affective component of perceived risk was more closely aligned with preventive compliance than perceived susceptibility to infection in this cohort. This pattern is broadly consistent with previous evidence showing that higher COVID-19 risk perception is generally associated with greater adoption of preventive behaviors and social-distancing measures. Similarly, studies among transplant recipients have linked greater COVID-19–related fear with selected precautionary behaviors, although the reported associations were modest (56, 57). SDHS scores were not significantly correlated with total COVID-RPS or fear perception. Although SDHS showed a weak negative correlation with contamination perception (rho = −0.125, *p* = 0.028) and a very weak positive correlation with COVID-PGCS (rho = 0.132, *p* = 0.020), the small magnitude of these associations limits their clinical relevance. Overall, preventive compliance was more consistently related to COVID-19 risk perception, particularly its fear dimension, than to SDHS-defined psychological well-being. Given the cross-sectional design and exploratory nature of these analyses, neither the direction nor the causal basis of these relationships can be determined.

Beyond these inter-scale correlations, the multivariable analysis identified several factors independently associated with SDHS scores. Recipients in the middle- and high-income groups had higher SDHS scores than those in the low-income group. This finding is broadly consistent with previous literature identifying socioeconomic disadvantage as an important correlate of poorer mental health during the COVID-19 pandemic (61). Everolimus use and reporting scientific journals and official documents as influential sources of COVID-19 information were also associated with higher SDHS scores. However, directly comparable evidence for these associations is limited, and they should therefore be considered exploratory and hypothesis-generating rather than evidence of beneficial effects of everolimus or scientific information exposure. In contrast, recipients who reported COVID-19 infection after BNT162b2 vaccination had lower SDHS scores than those who had never been diagnosed with COVID-19. This estimate should be interpreted with particular caution because the subgroup included only seven recipients. Residual confounding and unmeasured socioeconomic, clinical, and behavioral factors may partly explain all of these observational associations.

Findings from Türkiye provide additional context for the behavioral and psychological responses observed in clinically vulnerable populations. In a descriptive study of Turkish LT recipients, Doğan et al. (63) reported that higher COVID-19 fear scores were associated with less willingness to enter crowded environments, use public transportation, and attend medical appointments, together with greater reported increases in handwashing and mask or glove use. These findings suggest that COVID-19–related fear may be associated with both protective actions and avoidance of healthcare or social environments. In a separate cross-sectional study of Turkish patients with advanced HCC receiving non-transplant treatment or awaiting LT, Akbulut et al. (64) used a multilayer perceptron model in which chronic disease, sex, and age were among the most important variables for DASS-21 total scores. These studies provide relevant national context for the behavioral correlates of COVID-19–related fear and the clinical and sociodemographic characteristics associated with psychological distress. However, direct comparisons with the present findings are limited by differences in study populations, outcome measures, and analytical methods.

Evidence from other clinically vulnerable populations further illustrates the relationship between perceived infectious threat and psychological distress. Among people with multiple sclerosis, Alschuler et al. (65) found that greater anxiety was associated with higher perceived risks of contracting and dying from COVID-19. In an international study of patients with cancer, Cheli et al. (66) reported that higher COVID-19 risk perception was associated with greater psychosocial distress and that the strength of this relationship varied according to personality characteristics. Fadlallah et al. (67), although not directly assessing risk perception, found more severe anxiety—but not depressive symptoms—among SOT recipients who were transplanted and assessed after the onset of the pandemic than among a pre-pandemic reference group. Collectively, these studies suggest that perceived infectious threat and pandemic-related stress may be associated with anxiety and psychological distress in clinically vulnerable populations. However, because anxiety was not directly measured in the present study, our findings should be interpreted as demonstrating a weak-to-moderate association between COVID-19 risk perception, particularly its fear dimension, and preventive compliance, together with only a very weak positive association between SDHS-defined psychological well-being and compliance. These cross-sectional findings do not establish the direction or causal basis of the observed relationships.

Clinical characteristics, including immunosuppressive treatment, may provide additional context for the risk-perception findings. Evidence regarding COVID-19 outcomes after LT has varied across studies and pandemic periods. Kulkarni et al. (68) reported a cumulative mortality of 17.4% and severe COVID-19 in approximately 23% of infected LT recipients, but found no significant difference in mortality between LT and non-LT patients. More recent evidence across immunocompromised populations has generally indicated that SOT recipients remain at increased risk of severe COVID-19–related outcomes, although the magnitude of this risk varies according to organ type, age, comorbidity burden, vaccination response, pandemic period, and the type and intensity of immunosuppression (69). Among transplant recipients, Moazzam et al. (57) reported substantial COVID-19–related fear and high adherence to preventive measures. Higher fear was associated with selected precautionary behaviors, including mask use, avoidance of in-person clinic visits, and leaving shoes outside the home, although the reported effect sizes were small.

Related evidence from a non-transplant population suggests that immunosuppressant use may also be associated with perceived vulnerability. In a cross-sectional study of adolescents and young adults, Yang et al. (70) found that greater disease knowledge, chronic disease, and immunosuppressant use were independently associated with higher perceived risk of COVID-19 infection, with the strongest reported association observed for immunosuppressant use (aOR 2.53). In the present cohort, however, everolimus and prednisolone use were associated with lower total COVID-RPS scores only in exploratory univariate comparisons, and neither medication remained independently associated with total COVID-RPS in the multivariable model. These unadjusted differences may reflect treatment selection, comorbidity, time since transplantation, or other unmeasured clinical characteristics rather than medication-specific effects. Accordingly, the findings do not support interpreting individual immunosuppressive agents as independent determinants of COVID-19 risk perception.

In the full multivariable analysis of total COVID-RPS scores, recipients in the middle-income group had significantly lower scores than those in the low-income group (*B* = −1.520, *β* = −0.151, *p* = 0.011), whereas endorsement of protecting parents and children as a vaccination-related motivation was independently associated with higher total COVID-RPS scores (*B* = 1.813, *β* = 0.146, *p* = 0.040). These findings suggest that both socioeconomic circumstances and family-oriented protective motivations may be relevant to how LT recipients appraise COVID-19 risk. The lower risk-perception scores observed in the middle-income group should not necessarily be interpreted as a linear income gradient, particularly because the univariate findings indicated a more complex pattern across income categories. Similarly, the association with protecting parents and children may reflect a heightened sense of responsibility toward vulnerable family members rather than concern limited to the recipient’s own health.

### 4.1 Limitations

This study has several limitations. (i) Participants were consecutively recruited among clinically stable LT recipients attending routine outpatient follow-up visits. Therefore, selection bias cannot be excluded, and the study population may not fully represent recipients who do not attend follow-up regularly, are hospitalized, or have severe medical complications. The single-center setting further limits the generalizability of the findings to the broader LT population. (ii) A substantial proportion of participants had no formal education or only primary education, which may have affected their comprehension and interpretation of some questionnaire items. Although standardized face-to-face administration may have facilitated comprehension and reduced incomplete responses, interviewer-related influences and measurement error cannot be completely excluded. Furthermore, the use of self-reported data may have introduced recall and social desirability bias. (iii) The COVID-RPS demonstrated moderate internal consistency, lower than that of the SDHS and COVID-PGCS. Therefore, findings based on the COVID-RPS, particularly those involving its subdimensions, should be interpreted cautiously. (iv) Several vaccine acceptance– and hesitancy-related items had very small numbers of affirmative responses. These variables were therefore evaluated descriptively and were not included in the multivariable models, limiting the assessment of their independent associations with the study outcomes. In addition, only seven participants were included in the category of COVID-19 infection after BNT162b2 vaccination. Although this category was retained in the SDHS model, the corresponding estimate should be interpreted cautiously because of the small subgroup size and limited statistical precision. (v) The unequal sex distribution, with 238 male and 73 female participants, may have reduced the statistical power and precision of sex-specific comparisons. Sensitivity analyses showed that sex was not independently associated with SDHS, COVID-PGCS, or total COVID-RPS scores after adjustment for the variables included in the respective multivariable models (*p* = 0.890, *p* = 0.317, and *p* = 0.259, respectively). Although female participants had higher COVID-RPS fear-perception scores in the univariate analysis, this difference did not remain statistically significant after adjustment for the variables included in the multivariable model (*p* = 0.255). (vi) Numerous subgroup comparisons were performed, increasing the possibility that some nominally significant or borderline findings occurred by chance. Therefore, the univariate analyses should be regarded as exploratory, although Bonferroni correction was applied to relevant *post hoc* pairwise comparisons. (vii) In the total COVID-RPS model, COVID-19 vaccination status and infection-timing categories contained overlapping information. Their statistical significance changed when either variable was excluded in the sensitivity analyses, indicating model-specification dependence. Consequently, their individual associations with COVID-19 risk perception could not be clearly separated and should not be interpreted as robust, mutually independent effects. (viii) Although the overall COVID-PGCS model was statistically significant, none of the individual variables reached statistical significance, and the model explained only a small proportion of the variance. This indicates that potentially relevant determinants of preventive compliance may not have been fully captured by the variables examined in this study. (ix) The cross-sectional design precludes causal inference, and data collection during a specific phase of the COVID-19 pandemic may limit the applicability of the findings to other pandemic periods. Future multicenter and longitudinal studies with larger and more sex-balanced samples are needed to confirm these findings. Qualitative studies may further clarify the reasons underlying vaccination acceptance and hesitancy, while interventional studies may evaluate strategies to improve psychosocial well-being and preventive behaviors among LT recipients.

## 5 Conclusion

This study demonstrated a high rate of COVID-19 vaccination among LT recipients, reflecting substantial vaccine uptake in a clinically vulnerable population receiving long-term immunosuppressive therapy. COVID-19 risk perception and adherence to preventive behaviors were weakly to moderately related, suggesting partially overlapping but distinct constructs rather than a direct causal pathway. Higher SDHS scores were independently associated with middle income, high income, everolimus use, and influence of scientific journals and official documents, whereas COVID-19 infection after BNT162b2 vaccination was associated with lower SDHS scores. However, this latter finding should be interpreted cautiously because the subgroup included only seven recipients. No individual variable was independently associated with COVID-PGCS scores, despite the statistical significance of the overall model and its limited explanatory power.

In the full multivariable COVID-RPS model, middle income was independently associated with lower total COVID-RPS scores, whereas endorsement of protecting parents and children was associated with higher scores. Neither COVID-19 vaccination status nor the infection-timing categories were independently associated with total COVID-RPS scores in the full model. Sensitivity analyses showed that vaccination status became significant when the infection-timing categories were excluded, whereas infection before vaccination became significant when vaccination status was excluded. These findings indicate overlapping information and model-specification dependence; therefore, vaccination status and infection timing should not be interpreted as robust, mutually independent determinants of COVID-19 risk perception. Overall, the findings underscore the potential value of reliable scientific communication, psychosocial support, and sustained engagement with LT recipients during future infectious disease outbreaks. Further multicenter and longitudinal studies are needed to clarify the determinants of preventive behavior and to confirm the observed psychosocial and risk-perception associations.

## Data Availability

The raw data supporting the conclusions of this article will be made available by the authors, without undue reservation.
