# Supplementary material for: COVID-19 risk perception, preventive behaviors, vaccination acceptance and hesitancy, and psychosocial status in liver transplant recipients
Source: Front Public Health. 2026 Sep 4;14:1884917. doi: 10.3389/fpubh.2026.1884917 (PMC13585998; doi:10.3389/fpubh.2026.1884917)
Supplement: Supplementary file 1 [file Table_1.DOCX]

**Table S1**: COVID-RPS Contamination Perception Subscale Scores Across Sociodemographic, Clinical, Immunosuppressive, Vaccination-Related, and COVID-19–Related Variables

| Variable | Category | Mean | SD | 95% CI | p |
| --- | --- | --- | --- | --- | --- |
| Age group | ≤35 years | 18.1 | 3.3 | 17.1–19.1 | 0.390 |
|  | 36–45 years | 18.7 | 2.3 | 17.9–19.4 |  |
|  | 46–55 years | 17.6 | 2.7 | 17.0–18.3 |  |
|  | 56–65 years | 18.3 | 3.1 | 17.7–18.8 |  |
|  | ≥66 years | 17.9 | 2.6 | 17.0–18.8 |  |
| Sex | Male | 18.0 | 2.8 | 17.7–18.4 | 0.413 |
|  | Female | 18.4 | 3.0 | 17.7–19.1 |  |
| Educational attainment | No formal education | 17.1 | 2.7 | 15.9–18.4 | **0.019** |
|  | Primary education | 18.1 | 2.8 | 17.6–18.6 |  |
|  | Lower secondary education | 18.8 | 2.5 | 18.1–19.5 |  |
|  | Upper secondary education | 17.6 | 3.3 | 16.8–18.3 |  |
|  | Tertiary education | 19.0 | 2.6 | 18.2–19.9 |  |
| Marital Status | Married | 18.2 | 2.7 | 17.8–18.5 | 0.425 |
|  | Single | 17.8 | 3.5 | 16.8–18.9 |  |
| Monthly household income | Low income | 18.5 | 2.6 | 18.1–18.9 | **<0.001** |
|  | Middle income | 17.5 | 2.9 | 17.0–18.0 |  |
|  | High income | 19.8 | 3.1 | 18.3–21.3 |  |
| Employment Status | Employed | 17.8 | 2.6 | 17.0–18.5 | 0.364 |
|  | Unemployed | 18.2 | 2.9 | 17.8–18.5 |  |
| Chronic  Comorbidities | Present | 18.3 | 2.7 | 17.8–18.8 | 0.472 |
|  | Absent | 18.0 | 3.0 | 17.6–18.4 |  |
| HT | Present | 18.5 | 2.5 | 17.7–19.3 | 0.396 |
|  | Absent | 18.1 | 2.9 | 17.7–18.4 |  |
| DM | Present | 18.2 | 2.8 | 17.6–18.9 | 0.679 |
|  | Absent | 18.1 | 2.9 | 17.7–18.5 |  |
| CVD | Present | 19.1 | 3.1 | 17.4–20.8 | 0.188 |
|  | Absent | 18.1 | 2.9 | 17.7–18.4 |  |
| Everolimus use | Yes | 17.7 | 2.8 | 17.2–18.2 | **0.037** |
|  | No | 18.4 | 2.9 | 18.0–18.8 |  |
| MMF use | Yes | 17.8 | 3.2 | 17.0–18.5 | 0.217 |
|  | No | 18.2 | 2.8 | 17.9–18.6 |  |
| Prednisolone use | Yes | 17.5 | 3.2 | 16.7–18.2 | **0.021** |
|  | No | 18.3 | 2.7 | 18.0–18.7 |  |
| Have you ever received a COVID-19 vaccine? | I have | 18.0 | 2.9 | 17.7–18.4 | 0.280 |
|  | I have not | 18.5 | 2.7 | 17.8–19.3 |  |
| Type of COVID-19 vaccine received | CoronaVac | 17.9 | 2.9 | 17.4–18.5 | 0.692 |
|  | BNT162b2 | 18.0 | 2.9 | 17.4–18.6 |  |
|  | Heterologous vaccination | 18.3 | 3.0 | 17.6–19.0 |  |
| Have you ever had COVID-19? | I have | 17.5 | 2.7 | 17.0–18.0 | **0.008** |
|  | I have not | 18.4 | 2.9 | 18.0–18.8 |  |
| Timing of COVID-19 infection in relation to vaccination | Before vaccination | 16.7 | 2.7 | 15.9–17.5 | 0.086 |
|  | After CoronaVac vaccination | 18.1 | 2.8 | 17.0–19.2 |  |
|  | After BNT162b2 vaccination | 18.0 | 1.5 | 16.6–19.4 |  |
|  | Unvaccinated individuals with prior COVID-19 infection | 18.1 | 2.6 | 17.0–19.2 |  |

BNT162b2: Pfizer–BioNTech; CVD: Cardiovascular disease; COVID-19: Coronavirus disease 2019; CoronaVac: Sinovac; DM: Diabetes mellitus; HT: Hypertension; MMF: Mycophenolate mofetil; RPS: Risk Perception Scale

**Table S2:** COVID-RPS Fear Perception Subscale Scores Across Sociodemographic, Clinical, Immunosuppressive, Vaccination-Related, and COVID-19–Related Variables

| Variable | Category | Mean | SD | 95% CI | p |
| --- | --- | --- | --- | --- | --- |
| Age group | ≤35 years | 10.7 | 3.3 | 9.7–11.7 | 0.369 |
|  | 36–45 years | 11.1 | 2.5 | 10.4–11.9 |  |
|  | 46–55 years | 10.2 | 2.9 | 9.5–10.9 |  |
|  | 56–65 years | 10.9 | 3.0 | 10.3–11.4 |  |
|  | ≥66 years | 11.1 | 2.7 | 10.2–12.0 |  |
| Sex | Male | 10.6 | 3.1 | 10.2–11.0 | **0.030** |
|  | Female | 11.3 | 2.4 | 10.8–11.9 |  |
| Educational attainment | No formal education | 10.6 | 2.5 | 9.5–11.8 | 0.580 |
|  | Primary education | 10.6 | 2.8 | 10.1–11.1 |  |
|  | Lower secondary education | 11.2 | 3.0 | 10.3–12.0 |  |
|  | Upper secondary education | 10.6 | 3.2 | 9.9–11.3 |  |
|  | Tertiary education | 11.3 | 2.9 | 10.3–12.2 |  |
| Marital Status | Married | 10.9 | 2.9 | 10.5–11.2 | 0.130 |
|  | Single | 10.2 | 3.3 | 9.2–11.1 |  |
| Monthly household income | Low income | 11.1 | 2.8 | 10.7–11.6 | **0.035** |
|  | Middle income | 10.3 | 3.1 | 9.8–10.8 |  |
|  | High income | 11.4 | 2.6 | 10.1–12.6 |  |
| Employment Status | Employed | 9.8 | 3.1 | 8.9–10.7 | **0.019** |
|  | Unemployed | 10.9 | 2.9 | 10.6–11.3 |  |
| Chronic  Comorbidities | Present | 11.1 | 2.7 | 10.6–11.7 | 0.099 |
|  | Absent | 10.6 | 3.0 | 10.1–11.0 |  |
| HT | Present | 11.6 | 2.2 | 10.9–12.3 | **0.014** |
|  | Absent | 10.6 | 3.0 | 10.3–11.0 |  |
| DM | Present | 10.9 | 2.9 | 10.2–11.6 | 0.601 |
|  | Absent | 10.7 | 2.9 | 10.3–11.1 |  |
| CVD | Present | 11.0 | 2.8 | 9.5–12.5 | 0.741 |
|  | Absent | 10.7 | 2.9 | 10.4–11.1 |  |
| Everolimus use | Yes | 10.4 | 2.9 | 9.9–10.9 | 0.116 |
|  | No | 11.0 | 3.0 | 10.5–11.4 |  |
| MMF use | Yes | 10.3 | 3.2 | 9.5–11.1 | 0.317 |
|  | No | 10.9 | 2.8 | 10.5–11.3 |  |
| Prednisolone use | Yes | 10.3 | 2.7 | 9.7–10.9 | 0.113 |
|  | No | 10.9 | 3.0 | 10.5–11.3 |  |
| Have you ever received a COVID-19 vaccine? | I have | 10.7 | 3.0 | 10.4–11.1 | 0.851 |
|  | I have not | 10.8 | 2.8 | 10.0–11.7 |  |
| Type of COVID-19 vaccine received | CoronaVac | 10.5 | 3.1 | 9.9–11.1 | 0.231 |
|  | BNT162b2 | 10.7 | 2.7 | 10.1–11.3 |  |
|  | Heterologous vaccination | 11.2 | 3.0 | 10.5–11.9 |  |
| Have you ever had COVID-19? | I have | 10.3 | 2.8 | 9.8–10.8 | **0.051** |
|  | I have not | 11.0 | 3.0 | 10.6–11.4 |  |
| Timing of COVID-19 infection in relation to vaccination | Before vaccination | 9.4 | 2.7 | 8.5–10.2 | **0.039** |
|  | After CoronaVac vaccination | 11.0 | 3.0 | 9.9–12.1 |  |
|  | After BNT162b2 vaccination | 11.1 | 1.6 | 9.7–12.6 |  |
|  | Unvaccinated individuals with prior COVID-19 infection | 10.8 | 2.6 | 9.7–11.9 |  |

BNT162b2: Pfizer–BioNTech; CVD: Cardiovascular disease; COVID-19: Coronavirus disease 2019; CoronaVac: Sinovac; DM: Diabetes mellitus; HT: Hypertension; MMF: Mycophenolate mofetil; RPS: Risk Perception Scale
